# Investigating light sensitivity in bipolar disorder (HELIOS-BD)

**DOI:** 10.12688/wellcomeopenres.20557.2

**Published:** 2024-10-18

**Authors:** Amber Roguski, Nicole Needham, Tom MacGillivray, Jasna Martinovic, Baljean Dhillon, Renata L. Riha, Lyle Armstrong, Iain H. Campbell, Amy Ferguson, Gerrit Hilgen, Majlinda Lako, Philipp Ritter, Nayantara Santhi, Malcolm von Schantz, Manuel Spitschan, Daniel J. Smith

**Affiliations:** 1 Division of Psychiatry, The University of Edinburgh, Edinburgh, Scotland, UK; 2 Centre for Clinical Brain Sciences, The University of Edinburgh, Edinburgh, Scotland, UK; 3 Robert O Curle Ophthalmology Suite, Institute for Regeneration and Repair, The University of Edinburgh, Edinburgh, Scotland, UK; 4 Department of Psychology, School of Philosophy, Psychology and Language Sciences, The University of Edinburgh, Edinburgh, Scotland, UK; 5 Department of Sleep Medicine, Royal Infirmary of Edinburgh, NHS Lothian, Edinburgh, Scotland, UK; 6 Faculty of Medical Sciences, Newcastle University, Newcastle upon Tyne, England, UK; 7 Faculty of Health and Life Sciences, Northumbria University, Newcastle upon Tyne, England, UK; 8 Clinic for Psychiatry and Psychotherapy, Carl Gustav Carus University Hospital, Technische Universitat Dresden, Dresden, Saxony, Germany; 9 TUM School of Medicine & Health, Department of Health and Sport Sciences, Technical University of Munich, Munich, Bavaria, Germany; 10 TUM Institute for Advanced Study (TUM-IAS), Technical University of Munich, Munich, Bavaria, Germany; 11 Max Planck Research Group Translational Sensory & Circadian Neuroscience, Max Planck Institute for Biological Cybernetics, Tübingen, Germany

**Keywords:** Bipolar disorder, lithium, melatonin, light sensitivity, circadian rhythm, vision, sleep, retina

## Abstract

Many people with bipolar disorder have disrupted circadian rhythms. This means that the timing of sleep and wake activities becomes out-of-sync with the standard 24-hour cycle. Circadian rhythms are strongly influenced by light levels and previous research suggests that people with bipolar disorder might have a heightened sensitivity to light, causing more circadian rhythm disruption, increasing the potential for triggering a mood switch into mania or depression.

Lithium has been in clinical use for over 70 years and is acknowledged to be the most effective long-term treatment for bipolar disorder. Lithium has many reported actions in the body but the precise mechanism of action in bipolar disorder remains an active area of research. Central to this project is recent evidence that lithium may work by stabilising circadian rhythms of mood, cognition and rest/activity. Our primary hypothesis is that people with bipolar disorder have some pathophysiological change at the level of the retina which makes them hypersensitive to the visual and non-visual effects of light, and therefore more susceptible to circadian rhythm dysfunction. We additionally hypothesise that the mood-stabilising medication lithium is effective in bipolar disorder because it reduces this hypersensitivity, making individuals less vulnerable to light-induced circadian disruption.

We will recruit 180 participants into the HELIOS-BD study. Over an 18-month period, we will assess visual and non-visual responses to light, as well as retinal microstructure, in people with bipolar disorder compared to healthy controls. Further, we will assess whether individuals with bipolar disorder who are being treated with lithium have less pronounced light responses and attenuated retinal changes compared to individuals with bipolar disorder not being treated with lithium. This study represents a comprehensive investigation of visual and non-visual light responses in a large bipolar disorder population, with great translational potential for patient stratification and treatment innovation.

## 2 Introduction

This protocol is one part from a broader programme of work funded by the Wellcome Trust entitled “Lithium’s mechanism in bipolar disorder: investigating the light hypersensitivity hypothesis”. This protocol relates to the three University of Edinburgh workstreams (Parts A, B and C) of HELIOS-BD as follows:

Part A: Non-visual responses to light in bipolar disorder
Part B: Retinal sensitivity in bipolar disorder and lithium therapy
Part C: Retinal imaging in bipolar disorder and lithium therapy

There are two other components of the broader research programme investigating a) responses of bipolar disorder retinal organoids to light and lithium (basic wet-laboratory research) and b) the effect of lithium on non-visual response to light in people with no history of mental illness (clinical trial). These components will be described in separate protocol papers.

### 2.1 Circadian rhythms are disrupted in bipolar disorder

Bipolar disorder is a severe mood disorder affecting 1–2% of the global population ( Merikangas *et al.*, 2011). It is characterised by recurrent episodes of mania/hypomania and depression and is associated with a wide range of adverse health outcomes. Many of the clinical features of bipolar disorder – including extreme changes in mood, thinking, energy levels and rest/activity rhythms – can be conceptualised as fundamental disruptions of circadian rhythmicity ( McCarthy *et al.*, 2022). This project investigates one hypothesised cause of this circadian dysregulation (light sensitivity) and a potential protective mechanism of action for lithium.

### 2.2 Circadian rhythms are entrained to light

Circadian rhythms are endogenous near-24-hour rhythms which are continuously synchronised with the external light-dark cycle. The mechanism of this photic entrainment begins with photon absorption by intrinsically photosensitive retinal ganglion cells (ipRGCs) in the retina. This signals the presence of light to the hypothalamic suprachiasmatic nucleus (SCN), the core regulator of circadian rhythms. There are two main effects of photic signalling from the retina to the SCN. Firstly, light exposure acutely (and reversibly) suppresses the primary circadian-regulatory hormone, melatonin. Melatonin receptors are found throughout the central and peripheral nervous systems; thus, circulating melatonin levels signal external timings to the body. Secondly, light exposure at specific time windows has the ability to entrain the SCN clock by adjusting its phase. Given that the average free-running human circadian period is slightly above 24 hours, light in the morning is critical for maintaining circadian synchrony, whereas artificial light in the evening has the potential to significantly disrupt (or delay) it. A hypo- or hyper-sensitive response to light within the photoreceptive pathway can therefore result in a shifted circadian phase and dysregulation of biological rhythms.

There are a range of sensitivities to light within the general population, reflecting the varying influences of age, sex, pupillomotor function, and environmental light exposure upon photic response ( Chellappa, 2020; Swope *et al.*, 2023). However, light sensitivity has been shown to be enriched in particular populations, causing conditions such as delayed phase sleep wake disorder (DPSWD) ( Watson *et al.*, 2018) which are characterized by considerable circadian dysregulation. Light sensitivity has also been identified as a feature of several mood disorders ( Swope *et al.*, 2023) and thus represents an opportunity for improving understanding, treatment, and potentially prevention of these conditions.

### 2.3 Evidence for a light hypersensitivity in bipolar disorder

Since the 1980s, it has been hypothesized that photosensitivity to the non-visual effects of light could be the cause of the seasonal variation and circadian dysregulation seen in bipolar disorder populations. Disruption caused by hypersensitivity to light cues in bipolar disorder can lead to mood instability, disrupted sleep and ultimately episodes of hypomania, mania and/or depression (which themselves are characterised by circadian phase shifts ( Moon *et al.*, 2016)). Seasonal patterns in bipolar mood episodes provide clinical support for this theory, with hospital admission data showing trends for more mania-related admissions in spring/summer and more depression-related admissions in autumn/winter ( Geoffroy *et al.*, 2014). There is also an observed effect of seasonal changes in daily sunlight exposure on clinical outcomes such as suicide attempts ( Bauer *et al.*, 2021).

As both circadian and circannual rhythms are strongly influenced by light levels, a sensitivity to light within the photoreceptive pathway could be the cause of disruption to biological rhythms in bipolar disorder. Specifically, large changes in light levels - for example, rapid changes in photoperiod length at the spring and autumn equinoxes, or due to trans-meridian travel - may have a detrimental impact on circadian rhythms and bipolar disorder symptoms.

Experimentally, it has been shown that individuals with bipolar disorder exhibit greater light-induced phase delays ( Ritter *et al.*, 2021). Interventional studies in acute patient populations have also shown that blue-light blocking glasses may be effective in treating manic episodes ( Henriksen *et al.*, 2016; Hester *et al.*, 2021).

Melatonin suppression studies are the most direct way to test the causal effects of light on melatonin synthesis (and by proxy, circadian response to light). Several early studies showed that people with bipolar disorder have greater suppression of melatonin by light ( Hallam *et al.*, 2009; Lewy *et al.*, 1981; Lewy *et al.*, 1985; Nathan *et al.*, 1999), supporting a light hypersensitivity hypothesis. However, much of this research was conducted in small samples, and was conducted prior to the discovery of melanopsin in the late 1990s (thus missing a key piece of information regarding photoreceptive pathways). Several studies did not support a bipolar disorder light hypersensitivity ( Lam *et al.*, 1990; Whalley *et al.*, 1991), and although initial investigations suggested that light hypersensitivity might be a subpopulation feature of Type 1, but not Type 2, bipolar disorder ( Nurnberger *et al.*, 2000), more recent research into this hypothesis have called this into question ( Ritter *et al.*, 2020). To date, it has been difficult to establish clearly that light hypersensitivity is a feature of bipolar disorder because of poorly-controlled covariates within the small sample sizes reported in the literature. These limitations range from incomplete phenotyping of bipolar disorder participants, to a lack of measurement of pupil diameter and discrepancies in light delivery timing. Therefore, despite the intuitive potential of the light hypersensitivity hypothesis, current evidence is inconclusive.

Further, research investigating visual and non-visual responses to light have traditionally been siloed and there exists no complete theory of pathophysiological changes to the visual system in bipolar disorder which incorporates non-visual, circadian rhythm impacts. This is despite the knowledge that melanopsin-containing ipRGCs mediate visual-forming responses of retinal cone and rod cells ( Uprety *et al.*, 2022). Research has shown that people with bipolar disorder (or bipolar disorder traits) exhibit altered, state-dependent pupillary responses to light ( Bullock *et al.*, 2019; Madsen *et al.*, 2021) and reduced colour discrimination ( Fernandes *et al.*, 2017; Fernandes *et al.*, 2022; Garcia-Martin *et al.*, 2019) and visual processing ( Fernandes *et al.*, 2019), indicative of alterations in retinal sensitivity. Further to these functional changes, retinal imaging studies have found structural changes to the vasculature and retinal layers in individuals with bipolar disorder ( Appaji *et al.*, 2019; Garcia-Martin *et al.*, 2019; Prasannakumar *et al.*, 2023). It is not known whether, and how, these structural and visual function changes relate to non-visual responses in bipolar disorder.

### 2.4 A potential mechanism of action for lithium

Lithium has been in clinical use for over 70 years and is acknowledged to be the most effective long-term treatment for bipolar disorder. In the UK, the National Institute for Health and Clinical Excellence recommends lithium as a first-line treatment for bipolar disorder. However, lithium has variable efficacy within the bipolar disorder population, with roughly one third of individuals having a ‘full’ response. Lithium has many reported actions – including modulation of monoamine neurotransmission ( Ferrier *et al.*, 2006), inhibition of glycogen synthase kinase-3 ( Klein & Melton, 1996), reducing the hyperexcitability of neurons and neuroprotection ( Mertens *et al.*, 2015) – but the precise mechanism of action in bipolar disorder remains an active area of research. Given the considerable etiological and clinical heterogeneity of bipolar disorder, it is possible that lithium has different modes of action within different patient subgroups.

Central to this protocol is convergent evidence suggesting that lithium may work (at least in a proportion of patients) by stabilising aberrant circadian rhythms of mood, cognition and rest/activity. Animal studies demonstrate that lithium reduces light-induced phase delays ( Duncan *et al.*, 1998) and that it modulates the pupillary response to light ( Seggie *et al.*, 1989). In humans, 5 days of lithium ingestion was shown to significantly decrease melatonin suppression in healthy volunteers, with no significant effect on nocturnal melatonin secretion overall ( Hallam *et al*. 2005). Additionally, human MRI research showing that lithium accumulates in the eye ( Smith *et al*., 2018). Taken together, it is plausible that lithium’s circadian-regulating effects occur via an action at the level of the retina to reduce light sensitivity and non-visual response to light. If lithium were to normalise aberrant retinal signalling in some way, this may also reduce bipolar-specific visual responses to light. Further, sustained lithium use might be neuroprotective, preventing or attenuating progressive structural retinal changes.

### 2.5 HELIOS-BD study aims

The three-part HELIOS-BD protocol described here primarily seeks to investigate the light hypersensitivity hypothesis of bipolar disorder, with a mechanistic focus on the first component of the photoreceptive pathway: the retina. We hypothesise that retinal pathology causes light hypersensitivity in a proportion of people with bipolar disorder, which in turn increases susceptibility to circadian disruption and increases the risk of a mood episode. We will test whether structural and functional changes to the retina are progressive or stable with longitudinal data collection over 18 months. We will also test whether these bipolar-specific retinal changes cause changes to visual responses to light, using a range of psychophysical and electrophysiological methods. This project will therefore consolidate theories of visual and non-visual disturbances in bipolar disorder and will build an integrated picture of the bipolar disorder visual system.

These investigations will be expanded by incorporating lithium as a covariate and by assessing people with bipolar disorder either taking, or not taking, lithium. We hypothesise that lithium may be effective in bipolar disorder by acting at the level of the retina to reduce hypersensitivity to evening light. Specifically, we will assess visual and non-visual responses to light in people with bipolar disorder compared to healthy controls and, further, we will assess whether individuals with bipolar disorder who are being treated with lithium have responses to light that are attenuated compared to individuals with bipolar disorder not treated with lithium.

## 3 Protocol

### 3.1 Study objectives

The primary research question is ‘Do people with bipolar disorder have a hypersensitivity to visual and non-visual effects of light, and does lithium attenuate this hypersensitivity?’. This protocol is organized into 3 parts (Part A, B and C) to answer this question. The main objectives of each of these parts are as follows:

***3.1.1 Part A objectives***

1. To assess whether people with bipolar disorder (relative to controls with no history of psychiatric disorder) exhibit greater non-visual responses to light stimuli assessed by the light-induced suppression of melatonin secretion.
2. To assess whether these melatonin responses are less pronounced in those individuals with bipolar disorder who are being treated with lithium therapy (compared to individuals with bipolar disorder not taking lithium).

***3.1.2 Part B objectives***

1. To characterize the visual, cortical and pupillary responses of people with bipolar disorder (relative to controls with no history of disorder) using a combined electrophysiological and psychophysical study protocol.
2. To assess whether aberrant visual and non-visual responses are less pronounced in those individuals with bipolar disorder who are being treated with lithium therapy (compared to individuals with bipolar disorder not taking lithium).

***3.1.3 Part C objectives***

1. To assess whether people with bipolar disorder (relative to controls with no history of psychiatric disorder) exhibit microstructural changes in the retina.
2. To assess whether these changes are less pronounced in those individuals with bipolar disorder who are being treated with lithium therapy (compared to individuals with bipolar not taking lithium).

### 3.2 Public and patient involvement

***3.2.1 Agenda setting***

People with lived experience of bipolar disorder have been involved in the HELIOS-BD project from the very beginning. During the preparation of the funding application, two focus groups (Bipolar Scotland members) and 5 individual meetings with international people with bipolar disorder were conducted to gain insight into the research priorities and concerns of people with lived experience of bipolar disorder. During these discussions, topics of light perception, sleep, lithium and seasonality were explored in relation to bipolar disorder. It was found that these topics were relevant and of interest to people of bipolar disorder, with a consensus that mood changes were significantly influenced by the seasons, and a desire to understand this further. One focus of discussion was the efficacy of lithium at different times of the year, and the differing experiences of people with Type 1 and Type 2 bipolar disorder in relation to treatment, light perception and seasonal sensitivity.

***3.2.2 Study design***

The HELIOS-BD study design has been shaped by public and patient involvement during initial focus group discussions, study material review by members of Bipolar Scotland, and discussions with the newly-established HELIOS-BD Lived Experience Advisory Panel (LEAP) group following the funding award. Overall, our discussions with people with lived experience of bipolar disorder found people to be agreeable to the proposed study design.

Much of the focus of these discussions fell on Part A of the study, wherein participants attend 2 consecutive overnight stays at a clinical research facility and undergo serial blood sampling and a light exposure experiment. In response to concerns about the burden of sleep studies in relation to work schedules and responsibilities, the number of overnight stays was limited to the absolute minimum required to meet the study primary outcome measures (two nights). Altering light exposure during the study was considered to be an acceptable approach.

Feedback from members of Bipolar Scotland helped to ensure that participant-facing study materials, such as consent forms and Participant Information Sheets, were accessible and clear.

***3.2.3 Recruitment***

The HELIOS-BD LEAP group will act as ‘research champions’, making the bipolar disorder community in Scotland aware of this project and facilitating the dissemination of information about how to take part, via a social media campaign.

***3.2.4 Data collection, analysis and dissemination***

HELIOS-BD researchers and the HELIOS-BD LEAP group currently meet bi-monthly to ensure lived experience remains central to the project.

For the remainder of the project timeline, the research team and HELIOS-BD LEAP group will work closely together during the data collection, analysis and dissemination phases of the project. This collaboration will span a range of activities, including: consultation with the LEAP to troubleshoot and overcome relevant issues during data collection; working together to interpret results; conducting a community peer review to discuss study findings ( Liboiron *et al.*, 2018); determining different ways of communicating study results with relevant communities.

### 3.3 Study duration

The HELIOS-BD study will last for five years in total, with a start date of June 2023. We anticipate that screening and recruitment will begin in December 2023, and study visits will occur until January 2028 (t=54 months). This leaves 6 months for data analysis and dissemination.

### 3.4 Study design

This is an experimental study with cross-sectional and longitudinal data collection from three participant groups. Over an 18-month period, participants will attend multiple study visits to complete a range of tests, from retinal imaging to electrophysiological recordings and melatonin suppression experiments.

***3.4.1 Identification of potential participants***

**Figure 1.**
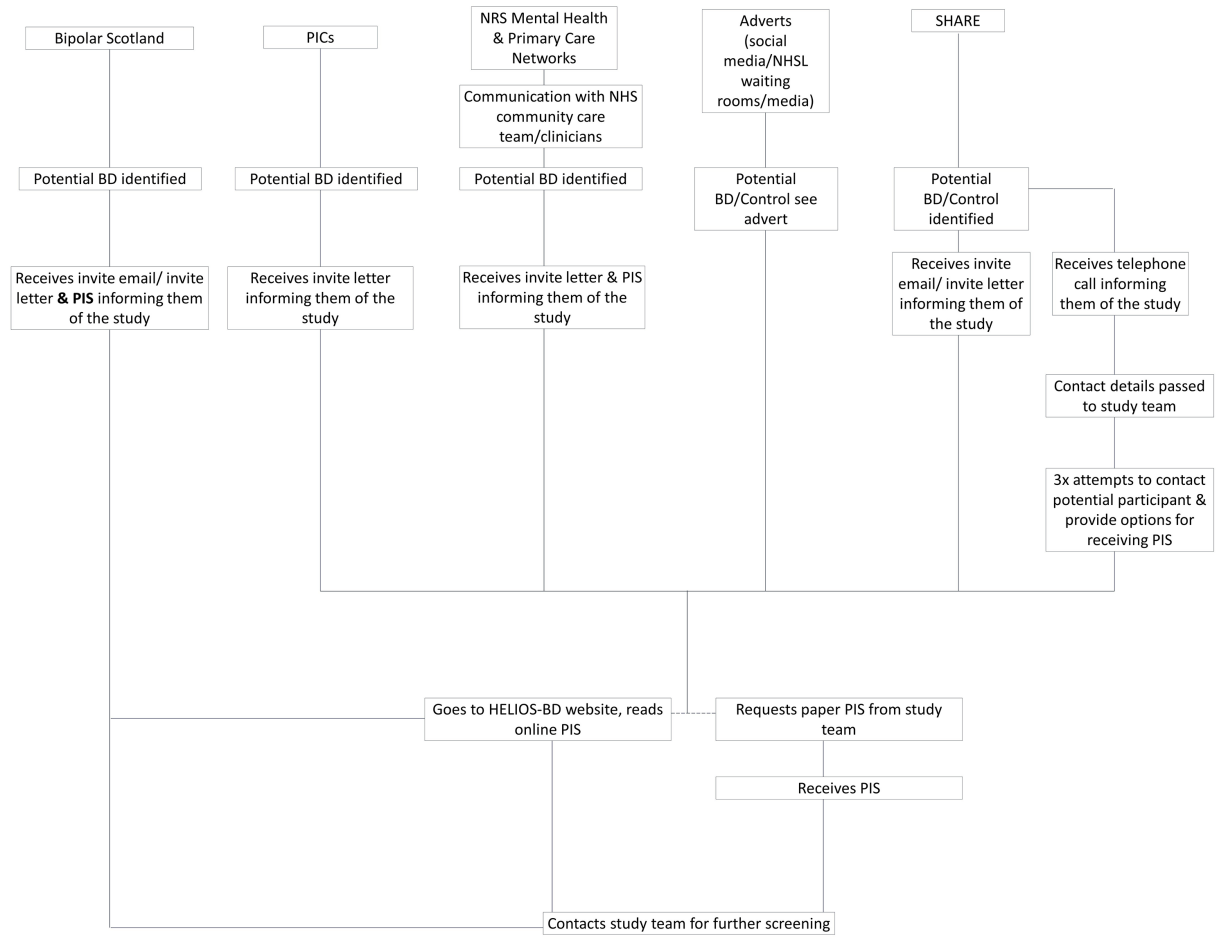
Overview of participant identification and recruitment procedures. Abbreviations: BD, bipolar disorder; NRS, NHS Research Scotland; PIS, Participant Information Sheet.

Our overall participant identification strategy represents a close collaboration with Bipolar Scotland, the Scottish Health Research Register (SHARE), the NHS Research Scotland (NRS) Mental Health Network and NRS Primary Care Network, and NHS health boards in Scotland who will act as Participant Identification Centres (PICs). An overview of potential participant identification and recruitment processes is given in Figure 1.

The primary recruitment avenue for both control and bipolar disorder groups will be via SHARE, a database of volunteers who consent to the access and use of their electronic health records to identify them for participation in research. SHARE will send an invitation letter and/or email with a link to the HELIOS-BD website (Participant Information sheet (PIS) available here) to potentially eligible participants, on behalf of the study team. There will also be an option for participants to request a paper copy of the PIS. These potential participants will then be able to contact the research team directly to express an interest. SHARE will also call some potential participants, using their web-based password-protected tracker to record these calls. The study team will be given the password and logs on to see who has been identified as wishing to take part, and to view any notes from the conversation, e.g., holiday dates, alternative number, working hours, etc. The study team will then directly contact those who expressed an interest by phone.

We will also recruit bipolar disorder participants from the NHS mental health services in Scotland via the NRS Mental Health Network. This network facilitates the recruitment of NHS patients into clinical research studies and clinical trials. The Network does this by working closely with NHS clinical teams to publicise studies to patients and clinicians through targeted advertising in clinical areas, and directly to the public. Where possible, the Network works with clinicians to identify potential participants, or pre-screens patient databases and reviews clinical records to create short lists of potential participants for clinical review. The clinical team can then inform these potential participants about the study and gain verbal consent for the NRS Mental Health Network to provide the PIS on behalf of the study team. The NRS Mental Health Network will also distribute the HELIOS-BD study poster to patient-facing locations.

Participants will additionally be recruited via the NRS Primary Care Network. This is a national, Scottish Government Health Directorate-funded network that facilitates eligibly funded research in primary care. Network staff work on behalf of the healthcare team under practice staff supervision. They sign practice confidentially agreements as required. In addition, each member of Network staff holds a current NHS substantive or honorary contract to allow them to carry out these duties. Searches are undertaken at each GP practice and the list generated by the search is screened by a clinician before invitation letters are sent out on behalf of the practice. Letters are sent out via docmail, an approved secure NHS mail service used by the Primary Care Network as standard. Patients are asked to contact the research team if they are interested in participating in the study. The network will recruit participants with bipolar disorder, but also control participants if required.

We will also list Scottish health boards as Participant Identification Centres (PICs), who will be able to identify potential research participants by processing personal data (they will carry out a search of patient record databases and caseloads to identify individuals that meet the studies eligibility criteria) and then send these potential participants a study information letter, with a link to the HELIOS-BD website (‘PIS available here’). These potential participants will then be able to contact the study team directly to express an interest, or to request a paper copy of the PIS if preferred.

Finally, we will recruit participants from the Bipolar Scotland ( www.bipolarscotland.org.uk) and Bipolar Edinburgh ( https://www.bipolaredinburgh.org.uk) membership across Scotland. Bipolar Scotland and Bipolar Edinburgh will help to publicise this study via social media and will send an invitation letter and PIS to their membership on behalf of the study team. Our Lived Experience Advisory Panel from Bipolar Scotland will act as ‘research champions’, making the bipolar disorder community in Scotland aware of this project and facilitating the dissemination of information about how to take part, via a social media campaign. Individuals who have been informed of the study either directly (through invite letter) or indirectly (through advertisement) will be invited to contact the study team via telephone or email for further information and screening.

***3.4.2 Screening procedure for recruitment***

Potential participants will have an initial screening call with a member of the study team to confirm that they meet the study inclusion criteria and to give them the opportunity to ask further questions about the study. The call will preferably be conducted using the ‘Nearme’ video consulting service on secure NHS Lothian accounts. If a potential participant does not have access to a smartphone or internet connection, the screening call will be conducted via telephone.

If the individual wishes to proceed with participation, personal data (age, sex (as defined by self-report), ethnicity, postcode (for socioeconomic status)) will be collected during this screening call. The researcher will complete a verbal consent form to gain consent from the participant to collect and securely store this demographic and eligibility data, and a copy of the verbal consent form will be sent to the participant. Their contact details will be recorded for study communication purposes and their participation appointment dates will then be booked.

***3.4.3 Consenting participants***

Consent will be taken from each participant and recorded on a consent form. Participants will be provided with a participant information sheet (PIS) at least 3 days prior to their initial screening telephone call, which will detail in full what they can expect from the study. They will be given an opportunity to discuss the study procedures during the initial consultation (via Nearme or phone), and have any questions answered. A summary of the research project will be provided by the research team member that will cover all elements specified in the PIS and consent form.

At their subsequent baseline study visit with a member of the research team, potential participants will be given the opportunity to ask questions and check their understanding of any points on the consent form before signing. It will be emphasised that the participant may withdraw their consent to participate at any time, without loss of any benefits to which they otherwise would be entitled.

The researcher taking consent will have undergone training in Good Clinical Practice. The principles of the Adults with Incapacity (Scotland) Act 2000 will also be followed. For the purposes of the Act, "incapable" means being unable to act on, make, communicate, understand, or retain the memory of decisions. If there are concerns that a potential participant’s capacity is borderline or likely to fluctuate, they will not be included in the study. All efforts will be made to avoid coercion and consent will be entirely voluntary, with potential participants allowed as much time as they need to decide. Potential participants will not have any identifiable dependent relationship to the research team.

All participants will be informed and will agree to their medical records being inspected by regulatory authorities and representatives of the sponsor(s). The Investigator or delegated member of the research team and the participant will sign and date the Informed Consent Form(s) to confirm that consent has been obtained. All participants will receive a copy of this document and a copy will be filed in the Investigator Site File (ISF) and sent to the participant’s GP.

***3.4.4 Participation procedures***

**Figure 2.**
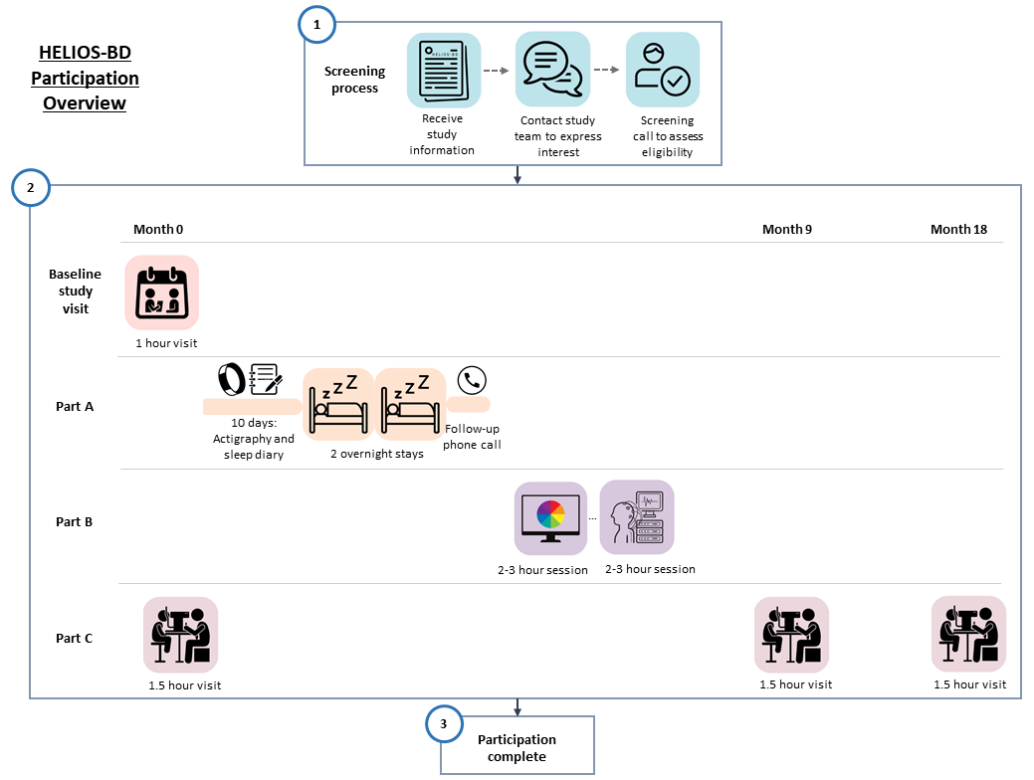
Overview of participation timeline and study procedures.

An overview of participation timeline and procedures is given in Figure 2.

**3.4.4.1 Baseline study visit**

Participant enrolment and baseline clinical assessment will be conducted in an appointment lasting up to 1 hour. All participants will be invited to attend this appointment face-to-face at the Clinical Research Facility (CRF) (Royal Infirmary of Edinburgh). Participants will be given the option to complete the baseline assessment in-person (at the Clinical Research Facility, CRF, RIE) or online via Near Me.

During this appointment, eligibility criteria will be checked again and individuals will give written consent to participate in the study. Written consent will either be on paper (if the appointment is in-person), or electronically (if the appointment is online). A structured clinical assessment will record:

- Details of demographics and lifestyle (educational attainment, employment status, socioeconomic status, smoking status (current smoker/non-smoker, ex-smoker, never smoked), substance use, and alcohol use assessed using the Alcohol Use Disorders Identification Test, AUDIT (Saunders *et al*., 1993))
- Sleep quality assessed using the Pittsburgh Sleep Quality Index (PSQI) ( Buysse *et al*., 1989) (licence agreement in place 04/03/24)
- Chronotype assessed using the Munich Chronotype Questionnaire (MCTQ) ( Roenneberg *et al*., 2003) (copyright license obtained 08/12/23)
- Global assessment of function (GAF) assessed using the WHODAS 2.0 ( Measuring Health and Disability, 2010; Ustün *et al*., 2010)
- Past psychiatric history (including age of onset, number and timing of episodes of depression, hypomania and mania, and a detailed assessment of past and current medication use, including the Retrospective Assessment of the Lithium Response Phenotype (Alda) Scale ( Grof *et al*., 2002).

**3.4.4.2 Part A: Overnight experimental assessments**

Two weeks prior to attending for the overnight experimental assessments, participants will be sent an actigraphy wristband and sleep diary, along with clear instructions for use (the actigraph can be preset to start recording when convenient). Daily light exposure and sleep-wake cycles will be monitored in the home setting for 10 days using the diary and wristband, as well as a record of daily caffeine and alcohol use. Participants will be given advice on limiting alcohol use, and on maintaining a regular sleep schedule for the duration of their participation.

In the rare occasion of a last-minute Part A cancellation, the appointment slot will be offered to an enrolled Control participant. In this instance, the actigraphy and sleep diary data will not be collected prior to the Part A overnight visits for that individual, and can be collected at another time point during the individual’s participation. This data is not required to meet the primary study outcomes.

Part A overnight experimental assessments will be conducted between the months of September-April inclusive to avoid the effects of too much light at night during the summer months.

Study Day 1

On Day 1, participants will attend the Clinical Research Facility in Edinburgh from 1700h. On arrival, there will be a check that participants still satisfy the inclusion and exclusion criteria and measurements of blood pressure, heart rate, height and weight will be taken. Participants will then be cannulated for the blood sampling used for melatonin measurements. A 2ml sample of blood will also be taken from the ‘bipolar disorder on Lithium’ group to test for lithium levels. An 8ml sample of blood will be collected and stored long-term for use in future ethically approved studies, including WP1 of the HELIOS-BD programme (wherein our collaborators in Newcastle/Northumbria will conduct retinal organoid research that is part of a separate ethics approval and protocol).

Participants will be required to ingest an eCelsius Performance telemetric temperature pill (BodyCAP, France) to monitor their core body temperature (CBT) throughout the first study night. The temperature-sensing pill is the size of a capsule tablet, does not record any information other than body temperature, and remains intact and will pass within 24–48 hours in the participant’s stool.

Participants will be offered meals as appropriate to the time of day and unlimited decaffeinated liquids. Participants will not be allowed to snack during the hours of blood sampling (19:00-06:00). From arrival at the CRF until lights out, participants will be kept under dim light conditions (<5 lux) until 2200h. During dim-light exposure, participants will not be allowed to use light-emitting devices such as mobile phones, tablets or e-readers. At 2200h, all lights will be turned off and participants will be invited to sleep. Participants can rise and turn on the lights at any time of their choosing from 0600h onwards.

During night 1, CBT will be measured with an ingestible eCelsius telemetric temperature pill, to allow us to schedule the light pulse during the subsequent night in reference to the temperature nadir. If the telemetric pill data cannot be collected (e.g. due to contraindication or data loss) then CBT nadir will be estimated using 12-day sleep diary. Blood sampling from a venous catheter will be performed hourly at night for baseline measurement of melatonin levels (1900h – 0600h). If at any point overnight it is no longer possible to use the cannula to obtain venous blood samples, up to 4 venous blood samples can be obtained via venepuncture. Samples will be centrifuged and blood plasma will be deep frozen until melatonin radioimmunoassay analysis.

Participants will be allowed to leave the Clinical Research Facility the next morning (Study Day 2) before 0900h, returning at 1700hr. Their venous cannula will be removed prior to their departure.

Study Day 2

On Study Day 2, participants will return to the CRF from 1700hr and will be subject to the same conditions as Study Day 1 (meals provided, unlimited decaffeinated liquids, no snacking, bed times and light exposure). Participants will be cannulated on arrival for the blood sampling used for melatonin measurements. During night 2, participants will be free to sleep from 2200h as they wish and are able to, except for the period of nocturnal light exposure for melatonin suppression when they need to stay awake with their eyes open.

Approximately 120 minutes before the CBT nadir, bilateral ocular application of Minims Tropicamide 1.0% will be carried out by a member of the research team to dilate the participant’s pupils. 90 minutes before the estimated CBT nadir, a member of the research team will expose participants to a 30-minute light stimulus. The light exposure will be delivered within a box measuring 100 × 80 × 50 cm. The box is painted white on the inside and contains an LED bulb, covered by a white shade with an aperture in the base. The front side of the box is open. Participants will be requested to keep their hands and head inside the box for 30-minutes, with eyes open throughout.

If the CBT measure is deemed to be inaccurate or unreliable (for example, in the case of data transmitting failure of the device to the receiver) the habitual mid-point of the participant’s sleep will be used as an indicator of the CBT nadir.

Blood sampling will be performed hourly at night to cover a period before and after the light exposure, for measurement of melatonin levels (1900h – 0600h). For the 90 minutes surrounding the light stimulation protocol (30 minutes prior, 30 minutes during, and 30 minutes post), blood samples will be collected at 15-minute intervals. If at any point overnight it is no longer possible to use the cannula to obtain venous blood samples, up to 4 venous blood samples can be obtained via venepuncture. Samples will be centrifuged and blood plasma will be deep frozen until melatonin radioimmunoassay analysis.

If the participant cannot complete the light stimulation protocol at the scheduled time due to an unforeseen issue (e.g. side effects from eye drops), the light stimulation protocol can be attempted/re-attempted within 1 hour of the scheduled time, at the discretion of the research staff, without needing to re-administer eye-drops. If light exposure is commenced but it is not possible to complete the full 30 minutes of exposure, the rest of the protocol procedures (e.g. blood sampling) will still be completed as planned (if possible). Light exposure will not be reattempted once the participant has been exposed to light, even if this is for a short period.

Study Day 3

On Study Day 3, participants can rise and turn on the lights at any time of their choosing from 0600h onwards. Their venous cannula will be removed and participants will be allowed to leave the Clinical Research Facility and go home. Due to the pupil dilation and disrupted sleep period, participants will be advised not to drive on Study Day 3 if they feel their vision has not returned to normal and it is unsafe to drive. Taxi transport will be provided if participants are unable to take public transport home.

After these assessments, all participants will be clinically assessed on the phone or via video call within 3 days to ensure that they have not experienced significant manic or depressive symptoms.

**3.4.4.3 Part B: Retinal assessments**

Experimental assessments will be conducted at the Colour and EEG laboratories (Dugald Stewart Building, University of Edinburgh). Testing will be completed in two sessions lasting approximately 2-3 hours each: one for colour psychophysics and pupillary responses; the other for visual evoked potentials. Participants will be assessed within a single day (with at least a 1 hour break in between, to avoid fatigue) or in 2 separate sessions, according to their preference. On arrival, there will be a check that participants still satisfy the inclusion and exclusion criteria.

Session 1 - Colour psychophysics and pupillary responses (Colour lab)

Participants will perform the following set of tasks:

1) Trivector test from the Cambridge Colour Test (Metropsis, Cambridge Research Systems, UK): circa 10 minutes (including instructions and some practice). The test measures three thresholds: along the 1) protan (Long-wavelength or L-cone), 2) deutan (Mid-wavelength or M-cone), and 3) tritan (Short-wavelength, or S-cone) axes. This test uses three interleaved adaptive psychophysical staircases to achieve a fast and precise measurement of cone function. It starts with a chromatic contrast at the extreme end of display gamut and subsequently modulates contrast along confusion lines that isolate L, M and S-cone function. Participants detect the orientation of a Landolt C type stimulus (up, down, left or right) embedded with a luminance-variegated background. Their responses control target chromatic contrast, so that the distance from neutral grey (i.e., background chromaticity) decreases for correct responses and increases for incorrect responses. This test has been used in a range of clinical populations (e.g., Parkinson’s disease, optic neuropathy, multiple sclerosis).

2) Contrast sensitivity functions (evaluated using Cambridge Research Systems, UK equipment) will provide a full characterisation of spatial vision driven by luminance and colour. In this test, gratings are displayed in the centre of the screen at 5 different spatial frequencies (range: 0.5 – 6 cycles per degree). Participants are asked to adjust the contrast of the stimulus until it is just barely visible. This should take 25 minutes in total, including instructions and some practice.

3) Suprathreshold just noticeable differences (JNDs) for luminance-defined, red-orange and blue will be measured using custom-made psychophysical procedures implemented on specialist colour vision equipment (CRS, UK). Participants will be presented with two stimuli, one on each side of the fixation cross. One of the two stimuli will be fixed (the standard stimulus; orange or turquoise) while the comparison stimulus will have its properties controlled by an adaptive staircase procedure. Participants will be instructed to report which of these two clearly visible (i.e., suprathreshold) stimuli is 1) brighter or 2) redder (for orange standard) or bluer (for turquoise standard). Manipulating the contrast of the comparison patch relative to the standard patch (which remains the same) allows for a precise measurement of a Just Noticeable Difference (JND) – the difference in luminance or chromatic contrast needed to make the two stimuli reliably different in appearance. The standard patch will be set to an intermediate hue (orange or turquoise) and at a luminance level brighter than the background, to mimic the appearance of disks in Lanthony’s D-15d test in which bipolar disorder participants have been reported to exhibit performance deficits ( Fernandes *et al.*, 2017). Administration takes ~20 minutes, including instructions and practice.

4) Pupil responses to light stimuli will be captured using a pupilometer capable of melanopsin-selective stimulation (OculoWise, Switzerland). Participants will first undergo 10 minutes dark adaptation before completing a post-illumination pupil response protocol, which measures pupil size in response to narrowband irradiance-matched red and blue light pulses. Similar protocols will then be administered using light pulses that (using the method of silent substitution) selectively target the cone-mediated luminance mechanism, and melanopsin. The resulting pupil responses are expected to provide a robust measure of increased retinal light sensitivity in bipolar disorder participants (administration ~45 minutes, including instructions and practice).

Session 2 - Visual evoked potentials (VEPs; EEG laboratory)

Participants will be able to take part in the EEG session either in the same afternoon (if they performed Session 1 in the morning) or on a separate day, depending on their preference.

Upon arrival, the EEG equipment will be set up and the electrodes positioned on the participant’s scalp. A BioSemi ActiveTwo (BioSemi, Netherlands) 64-electrode system will be used. Hypoallergenic electroconductive gel will be used to establish contact between the scalp and the electrode. Gel ensures a stable signal over a relatively long period of time.

We will measure VEPs elicited by contrast modulated patterns defined along the three retino-geniculate mechanisms (achromatic and chromatic). More specifically, pattern-onset and steady-state VEPs will be measured at multiple levels of contrast for uniform unipolar Gabor patches defined by increments and decrements along the achromatic, reddish-greenish and bluish yellowish axes. The experimental protocol will last for 1 hour.

EEG set up and removal will take approximately 60 minutes. An additional half-hour will be added to testing time to allow for participants to wash their hair in the laboratory’s hair-washing facilities after the data collection has ended, in order to remove the electroconductive gel.

**3.4.4.4 Part C: Retinal imaging**

Part C involves three study visits to image the microstructure of the retina and to investigate longitudinal retinal changes over an 18 month period. The schedule for Part C study visits is 0-, 9- and 18-month time-points. Each imaging session will last about up to 1.5 hours.

Imaging will be conducted at the Edinburgh Imaging facility in the Queen’s Medical Research Institute on the BioQuarter Little France campus. Retinal scanning is non-invasive and a completely safe method of obtaining pictures of the retina. Images are fast to acquire, and non-contact, non-invasive and require only simple chin-rest positioning. Pupil dilation is not required for any of the imaging.

A trained member of the research team will image both eyes of a participant using:

1. Fundus camera photograph: A fundus camera generates a 2D, high resolution image showing the optic disc, macula and blood vessels, and permits analysis of these anatomical features.
2. Ultra-widefield scanning laser ophthalmoscopy: An ultra-widefield scanning laser ophthalmoscope (UWF-SLO) captures more of the retina in one go ( ^~^80%) and facilitates the study features and changes in the periphery (e.g. prevalence of drusen, and vascular abnormalities and integrity), though at the sacrifice of a lower resolution for an increase in image field.
3. Optical coherence tomography (OCT): OCT images the retinal nerve fibre layer (RNFL) and inner layers of the retina.
4. OCT Angiography (OCT-A): OCT-A reveals the very small capillary level vessels in the retina which are not possible to image in any other way.
5. Optical biometry: Optical biometry yields the eye’s axial length - i.e. the distance from front to the back - a vital measure used to account for differences in individuals’ eyes and an important metric for the evaluation of retinal biomarkers.
6. Automated perimetry: Automated perimetry tests a participant’s visual field. The participant sits in front of the machine and presses a button when they see the presentation of a white light stimulus in various locations of their visual field. This will reveal any functional changes over time which can then be compared to any structural changes detect with the other imaging techniques.
7. Autorefraction: Autorefraction measures a participant’s refractive error (i.e. how short- or long-sighted they are).

Modes 1–4 of retinal scanning provide different but highly informative images of the back of the eye. Anonymised image data will be analysed by the relevant members of the research team in the Centre for Clinical Brain Sciences at the University of Edinburgh. There is a small risk that incidental findings may be identified on the retinal images which may require further investigation. We anticipate the rate of significant or urgent findings to be <1%. Each participant will be told of this risk, as well as it being stated on the participant information sheet. Should we observe unusual signs in the images that might signs of eye problems, anonymised images will be reviewed by the ophthalmologist on our team for checking of incidental findings. If we were to observe any such findings then an appropriate member of the study team would discuss this with the participant and inform their GP so that appropriate further tests and treatments could be arranged as necessary.

### 3.5 Data collection/participation windows

Baseline, Part B and Part C study visits and data collection will take place throughout the year.

Part A participant appointments and data collection will take place between the months of September and April (inclusive). Restricting data collection to these months avoids days with extremely long photoperiods (summer months) where night-time is limited or absent.

In the event of an abandoned study visit where a participant is unable to complete all protocol tasks, they will be invited back to complete the appointment at a later date if deemed appropriate by the study team.

The first screening and study visits (December 2023–March 2024) served as a pilot phase to fine-tune the assessments and/or their delivery. Protocol amendments were introduced where necessary

### 3.6 Study population

***3.6.1 Sample size calculation***

This study will assess 180 participants:

- *n*=60 with bipolar disorder and currently on lithium therapy
- *n*=60 with bipolar disorder and not taking lithium
- *n*=60 healthy controls with no history of mental illness and no first-degree family history of mental illness

The required sample size for this study is calculated from melatonin measures relevant to Part A. This sample size is sufficient for both Part B and Part C of the project (see below). Sex and gender differences were not taken into consideration in study design or sample size calculations because there is no conclusive evidence of sex or gender differences in the primary study outcome measures.

**3.6.1.1 Part A Sample Size Calculation**

Our subjects of interest are BD patients versus controls, with the BD group sub-divided into two groups: one where BD subjects are on lithium therapy and another where BD subjects are not taking lithium. Based on previous reports of light-induced melatonin suppression in BD ( Hallam *et al.*, 2009) we expect a difference of approximately 40–50% (Cohen’s d effect size 0.5–0.6) between the BD lithium group and the BD non-lithium group. As such, for 80% power with an alpha of 5%, we will need 42 participants in each of the three groups. Allowing for incomplete data collection in up 30% of participants, this means that we will recruit 60 participants in each group.

**3.6.1.2 Part B Sample Size Calculation**

With this sample size (N=180 participants across three groups), a two-sided between-subjects t-test at 80% power with an alpha level of 0.05 will be able to reliably detect an effect size of d=>0.52, equivalent to a difference of 0.52 of a standard deviation (i.e., medium effect size). Comparably, a between-subject ANOVA with three groups would be able to detect an effect size of f=>0.23. For a clinical study of this kind, this represents a very high level of sensitivity. As a minimum, it will allow us to provide a more definitive characterisation of the observed phenomena than previous studies in this field. As a comparison, studies with groups of 20 participants, which are common in the existing literature, are capable of detecting an effect of d=>0.9 (two groups) or f=>0.41 (three groups), which are large effect sizes.

**3.6.1.3 Part C Sample Size Calculation**

Part C is an exploratory study and is configured as the first step into investigating what sort of longitudinal retinal changes can be expected in a BD population. It is therefore difficult to estimate measurable effect size and very hard to predict which features will be discovered as relevant by computational approaches. However, the results of our analysis will be vital to informing future work. The samples size for Part C are thus informed by those of Part A and B, and an *n*=60 per group is used as an achievable target for imaging a sufficient number of participants for developing, testing and assessing our methods.

***3.6.2 Inclusion criteria***

**All participants**

Study participants must be adults ≥ 18 years old with capacity to give informed consent and to comply with the study design. All participants must be living in Scotland, be registered with a GP within NHS Scotland, and must have fluency in written or spoken English.

**BD Lithium participant group**

Participants in the ‘BD Lithium’ group must have a diagnosis of bipolar disorder according to DSM V criteria, confirmed with the Structured Clinical Interview for DSM5 (SCID) with at least one previous manic or two previous hypomanic episodes. Where possible, the diagnosis of bipolar disorder will be corroborated using clinical notes or records. Participants in the ‘BD Lithium’ group must be clinically euthymic with no episodes of depression, hypomania or mania for at least three months prior to enrolment. Participants will be asked to inform the study team of any mood episodes they have developed in the three months prior to any study appointment. Affected appointments will be rescheduled once the participant has been euthymic for 3 months. They must be on a stable lithium medication regimen for at least six months prior to study participation.

**BD Non-Lithium participant group**

Participants in the ‘BD Non-Lithium’ group must have a diagnosis of bipolar disorder according to DSM V criteria, confirmed with the Structured Clinical Interview for DSM5 (SCID) with at least one previous manic or two previous hypomanic episodes. Where possible, the diagnosis of bipolar disorder will be corroborated using clinical notes or records. Participants in the ‘BD Non-Lithium’ group must be clinically euthymic with no episodes of depression, hypomania or mania for at least three months prior to enrolment. Participants will be asked to inform the study team of any mood episodes they have developed in the three months prior to any study appointment. Affected appointments will be rescheduled once the participant has been euthymic for 3 months.

Participants in the ‘BD Non-Lithium’ group must not be currently taking lithium, and must have no lithium use for at least six months prior to study participation.

***3.6.3 Exclusion criteria***

**All participants**

Exclusion criteria for all participants includes the inability to give consent and inability to comply with study design. Individuals cannot participate in the study if they are currently enrolled in an interventional research study. Medical exclusion criteria include: pregnancy, diagnosis of sleep or circadian disorders; claustrophobia; epilepsy; blindness or significant visual impairments; existing eye disease such as glaucoma or age-related macular degeneration; previous surgery on the retina; use of melatonin or melatonin-suppressing medications (such as beta-blockers). Other significant medical conditions which, in the investigator’s opinion, could skew or confound data from investigations (e.g. neurological conditions such as multiple sclerosis) will be assessed on a case-by-case basis and excluded if necessary. Individuals with harmful use of or dependence on psychoactive substances, including alcohol (as per ICD-10) will not be eligible to participate. Due to their impacts on circadian rhythmicity, current shift work (variable day shift pattern or night shifts) and trans-meridian travel within the preceding month exclude individuals from participation.

**Control participant group**

Participants in the control group must not have recent history of any psychiatric illness or psychotropic medication use (within the past 12 months for psychiatric condition), or previous history of severe mental illness (psychosis, severe depression, psychiatric hospital admission). First-degree family history of psychosis or severe mood disorder excludes individuals from participation.

***3.6.4 Participation window***

The amount of time each participant will be actively participating in the study will vary depending on how closely together they wish to complete all parts. The anticipated minimum amount of time required will be 20 months, from receipt of study invite to completion of all assessments.

***3.6.5 Payment of Study Participants***

All enrolled study participants will be reimbursed for their participation time, costed at £15 per hour (estimated 38 hours total). If a participant withdraws from the study, they will be paid for the time they have completed up until that point. Travel (public transport, fuel, parking) and childcare costs will additionally be reimbursed. If a participant completes all of the assessments, they will be reimbursed up to a maximum of £750 including expenses. Payments will be made via bank transfer using University of Edinburgh standard procedure.

***3.6.6 Withdrawal of Study Participants***

Participants will be informed via the PIS that their participation is entirely voluntary and that they are free to withdraw from the study at any time. Information already collected will still be used when preparing the results of the study, unless the participant tells us they don’t want their information to be used in this way. Once the study has finished and the data have been analysed for publication, participants will no longer be able to withdraw their information. If a participant lost capacity during the study, it would not be appropriate to continue the study data collection as ongoing consent would be required. Data already collected would still be used. To safeguard rights, minimal personally identifiable information will be collected.

***3.6.7 Risk assessment and distress procedure***

The research team will have regular contact with participants in this study. If any significant issues arise, all participants will be informed by phone and letter. This includes both self-reported issues and issues that may arise from the tests conducted, or data collected. Some participants may find some questions in the questionnaires sensitive in nature (questions about their mental health past and present). Where there is any clinical risk to the participant (for example the disclosure of suicidal thoughts) the interviewing psychiatrist or research team member will ask for consent to contact the patient’s GP and treating community mental health team (if they have one), so that appropriate monitoring and follow-up can be arranged.

### 3.7 Study assessments

Table 1 details provides an overview of all study assessments and time points at which they will occur. Further details of the assessments are as follows:

**Table 1.** Study Assessment measures and collection time points. Abbreviations: AUDIT, Alcohol Use Disorders Identification Test; DSM-V, Diagnostic and Statistical Manual of Mental Disorders 5 ^th^ Edition; EEG, electroencephalography; OCT, Optical Coherence Tomography; OCT-A, Optical Coherence Tomography Angiography; m-SPAQ, Modified Seasonal Pattern Assessment Questionnaire; MCTQ, Munich Chronotype Questionnaire; VEPs, Visual Evoked Potentials.

Assessment	Screening Call	Baseline Clinical Assessment	Part A	Part B	Part C
1. Collection of contact details	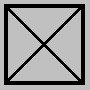	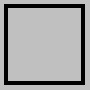	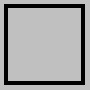	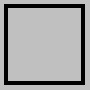	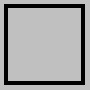
2. Verbal informed consent	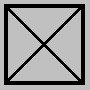	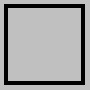	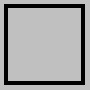	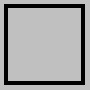	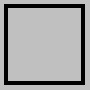
3. Assessment of Eligibility Criteria	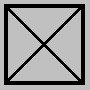	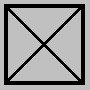	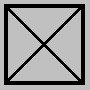	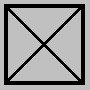	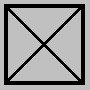
4. Written informed consent	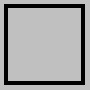	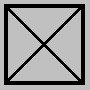	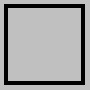	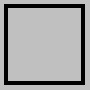	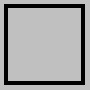
5. Demographic and lifestyle data	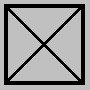	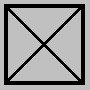	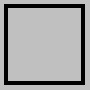	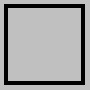	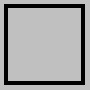
6. Alcohol Use Disorders Identification Test, AUDIT	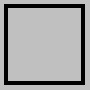	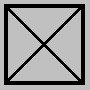	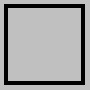	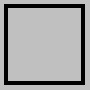	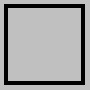
7. Past psychiatric history (including medical notes screening if possible)	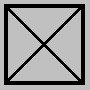	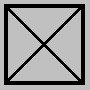	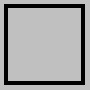	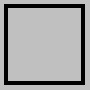	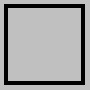
8. Assessment of past and current medication use	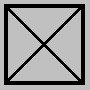	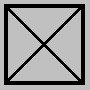	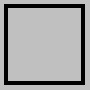	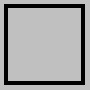	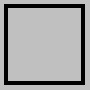
9. Retrospective Assessment of the Lithium Response Phenotype (Alda) Scale	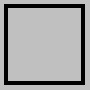	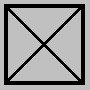	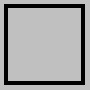	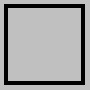	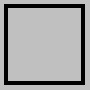
10. Pittsburgh Sleep Quality Index (PSQI) questionnaire	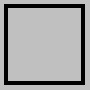	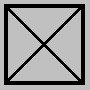	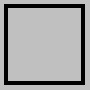	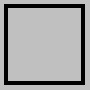	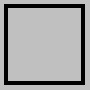
11. Munich Chronotype Questionnaire (MCTQ)	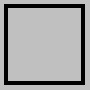	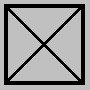	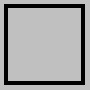	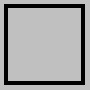	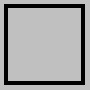
12. Modified Seasonal Pattern Assessment Questionnaire (m-SPAQ)	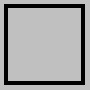	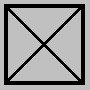	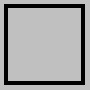	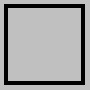	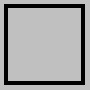
13. WHODAS 2.0 Questionnaire	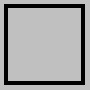	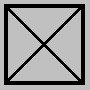	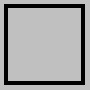	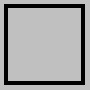	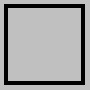
14. Sleep Diary	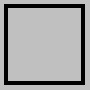	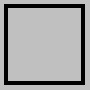	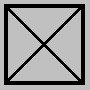	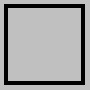	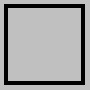
15. 24-hour actigraphy	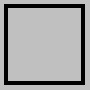	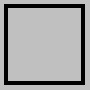	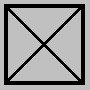	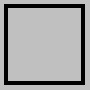	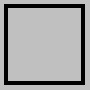
16. Measurement of light exposure levels	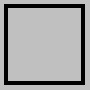	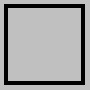	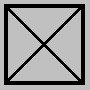	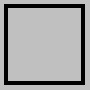	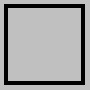
17. Pregnancy test (if applicable)	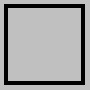	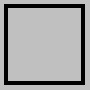	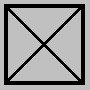	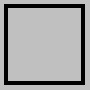	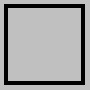
18. Heart rate, blood pressure, height and weight measurements	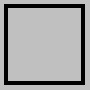	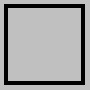	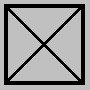	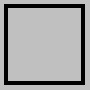	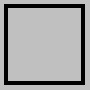
19. Cannulation	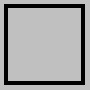	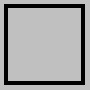	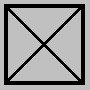	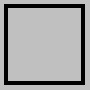	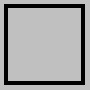
20. Blood sample collection	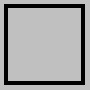	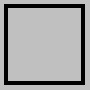	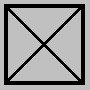	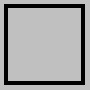	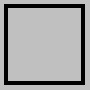
21. Ingestion of eCelsuis telemetric temperature pill	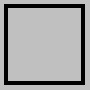	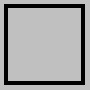	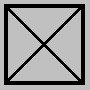	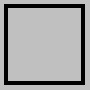	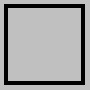
22. Overnight Sleep Study Protocol	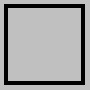	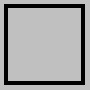	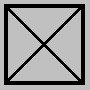	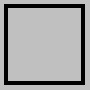	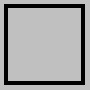
23. Bilateral ocular application of pupil dilator medication Minims Tropicamide 1.0%	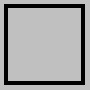	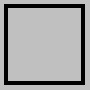	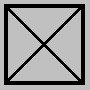	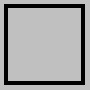	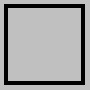
24. 30 minute light stimulus	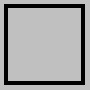	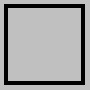	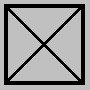	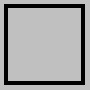	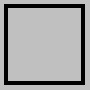
25. Follow-up wellbeing call	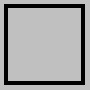	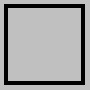	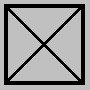	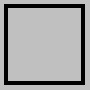	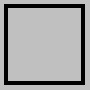
26. Cambridge Colour test	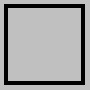	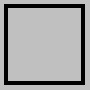	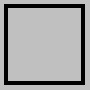	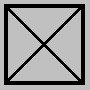	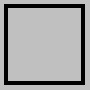
27. Contrast sensitivity test	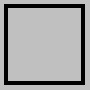	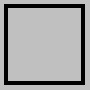	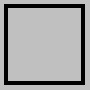	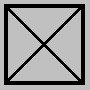	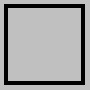
28. Colour discrimination test (Just noticible differences)	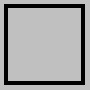	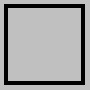	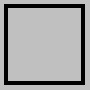	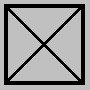	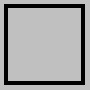
29. Pupillometry	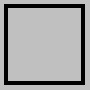	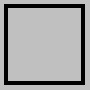	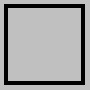	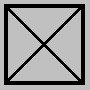	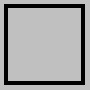
30. Electroencephalography (EEG)/ Visually evoked potentials (VEPs)	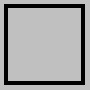	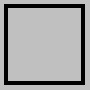	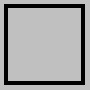	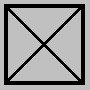	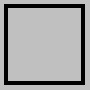
31. Fundus camera photograph	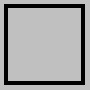	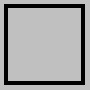	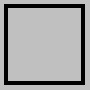	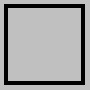	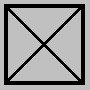
32. Ultra-widefield scanning laser ophthalmoscopy	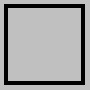	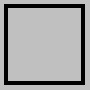	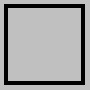	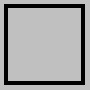	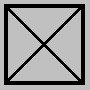
33. Optical coherence tomography (OCT)	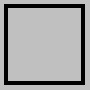	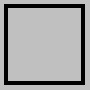	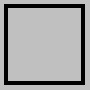	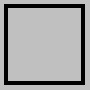	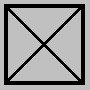
34. OCT Angiography (OCT-A)	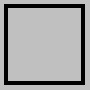	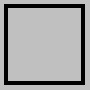	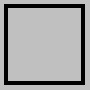	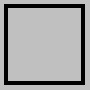	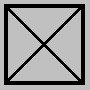
35. Optical Biometry	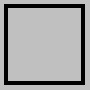	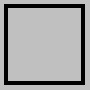	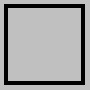	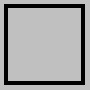	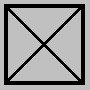
36. Automated perimetry	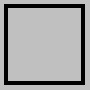	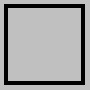	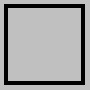	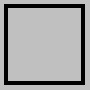	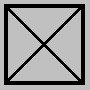
37. Autorefraction	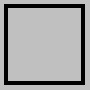	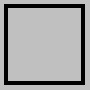	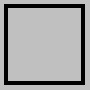	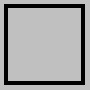	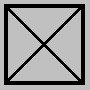

**3.7.1.1 Screening Appointment**

**1.** *Collection of contact details*: Contact details will be collected during the initial screening appointment to allow for further communications between the research team and potential participants.

**2.** *Verbal informed consent*: Potential participants will provide verbal informed consent for the purposes of the screening call data collection.

**3.** *Assessment of eligibility criteria*: Potential participants will be screened for eligibility criteria at the initial screening day and again at baseline study visit. This will include a structured diagnostic assessment for bipolar disorder at the screening appointment.

**3.7.1.2 Baseline study visit**

**4.** *Written informed consent*: Potential participants who meet the study eligibility criteria will provide written informed consent during enrolment to the study at baseline study visit.

**5** *Demographic and lifestyle data (self-reported)*: Age; self-reported sex; gender identity, ethnicity; educational attainment; employment status; socioeconomic status measured using the Townsend Deprivation Index; smoking status.

**6.** *Alcohol Use Disorders Identification Test, AUDIT (Saunders *et al*., 1993)*: Validated questionnaire to assess alcohol use.

**7.** *Past psychiatric history*: Data including age of onset, number and timing of episodes of depression, hypomania and mania will be collected. This is to gain a full understanding of the participant’s clinical history.

**8.** *Assessment of past and present medication use*: To be used as variates in future analyses.

**9.** *Retrospective Assessment of the Lithium Response Phenotype (Alda) Scale ( Grof *et al*., 2002)*: Validated questionnaire to retrospectively assess lithium response phenotypes in bipolar disorder participants.

**10.** *Pittsburgh Sleep Quality Index (PSQI) ( Buysse *et al*., 1989)*: Validated questionnaire which assesses sleep quality and sleep disturbance.

**11.** *Munich Chronotype Questionnaire (MCTQ) ( Roenneberg *et al*., 2003)*: Validated questionnaire to assess chronotype, collecting information about sleep and wake under natural conditions, including sleep and wake timings.

**12.** *Modified Seasonal Pattern Assessment Questionnaire (m-SPAQ) (modified from Rosenthal *et al*., 1984)*: Validated questionnaire to assess impact of seasons on mood and day-to-day life. The questionnaire is modified with the addition of several questions relating to bipolar disorder symptoms and seasonality, the results of which will not be aggregated into the SPAQ scoring system.

**13.** *WHODAS 2.0 Questionnaire ( Measuring Health and Disability, 2010; Ustün *et al*., 2010)*: Validated assessment instrument for health and disability, covering six domains of functioning: cognition, mobility, self-care, getting along, life activities, and participation.

**14.** *12-days of Sleep Diary*: For the 10 days prior to the overnight CRF study, and for the duration (2 days) of the overnight CRF study, participants will be required to complete a sleep diary to collect data on their sleep and wake timings.

**15.** *12-days of 24-hour actigraphy*: For the 10 days prior to the overnight CRF Study, and for the duration (2 days) of the overnight CRF study, participants will be required to wear a wristband actigraphy device (Axivity, UK) to measure their wake and sleep rhythms and wake activity levels.

**16.** *12-days of daily light exposure levels* (including daylight exposure on first study day): For the 10 days prior to the overnight CRF Study, and for the duration (2 days) of the overnight CRF study, estimates of light exposure will be derived from the AX3 actigraphy device (Axivity, UK) and ActLumus light logger (Condor Instruments, Brazil) respectively.

**3.7.1.3 Part A**

**17.** *Pregnancy test*: Upon arrival to the Part A appointment, participants of childbearing potential (between the stages of menarche and menopause) will be required to complete a pregnancy test to confirm that they are not pregnant prior to their ingestion of the telemetric pill.

**18.** *Heart rate, blood pressure, height and weight measurement*

**19.** *Cannulation:* Venous cannulation of hand or arm for blood sampling over the two nights.

**20.** *Blood sample collection:* A maximum of 31 blood samples (approximately 60mls total) will be taken over the course of the overnight stay:

1 sample: for lithium level testing (2mls) (only BD on Lithium group)
1 sample: for retinal organoid research (Helios-BD Work package 1) (8mls, EDTA Tube), if the participant has consented to their blood samples being used in future ethically approved studies
12 samples: twelve blood samples (2.5mls each) taken at hourly intervals between the hours of 7pm and 6am inclusive on Part A Study Day 1
17 samples: twelve blood samples (2.5mls each) taken at hourly intervals between the hours of 7pm and 6am inclusive on Part A Study Day 2. An additional five blood samples will be taken at 15 minute intervals around the light stimulation protocol (30 minutes prior, 30 minutes during, 30 minutes after).

**21.** *Ingestion of eCelsius telemetric temperature pill:* To measure core body temperature over 10 hours on the night of Study Night 1 for calculation of core body temperature nadir.

**22.** *Overnight Sleep Study Protocol:* Participants will be required to complete 2 sequential overnight stays at the Clinical Research Facility, Royal Infirmary of Edinburgh. During the Overnight stay, participants will be exposed to dim light conditions (<5 lux) from arrival until 2200h, and dark conditions between the hours of 2200h and 0600h.

**23.** *Bilateral ocular application of pupil dilator medication Minims Tropicamide 1.0%:* Application of pupil dilator solution will take place on Study Day 2 approximately 30 minutes prior to the light stimulation protocol

**24.** *30 minute light pulse*: Participants will be exposed to a 30 minute light pulse on Study Day 2. The light stimulation will be delivered 90 minutes prior to the estimated core body temperature nadir.

**25.** *Follow-up wellbeing call:* For participant wellbeing to ensure no experiences of significant manic or depressive symptoms occurred due to the study protocol. The follow-up assessment will consist of a telephone call from the clinical research fellow on the team within 3 days of the completion of Study Day 2.

**3.7.1.3 Part B**

**26.** *Cone-isolating chromatic thresholds*, measured using the Cambridge Colour Test. Participants observe Landolt Cs and indicate their orientation. Their responses feed into a staircase that controls stimulus contrast until a threshold is accurately measured.

**27.** *Contrast sensitivity functions*. Participants observe gratings of different spatial orientations and indicate their orientation. Their responses feed into a staircase that controls stimulus contrast until a threshold is accurately measured.

**28.** *Just noticeable differences for colour and luminance-defined stimuli* (blue, orange and luminance-defined). Participants observe two patches and indicate which one is brighter, redder or bluer. Their responses feed into a staircase that controls stimulus contrast until a threshold is accurately measured.

**29.** *Pupillary responses*. Participants are presented with lights that differentially stimulate melanopsin and chromatic pathways and their pupillary response is measured using a RetinaWise device from Oculowise (Switzerland).

**30.** *Visual evoked potentials to spatiochromatically modulated stimuli*. Participants will observe a series of bipolar Gabor patches defined along blue-yellow, red-green or achromatic dimensions at several levels of contrast - from low contrast to contrast matching the maximum achievable chromatic modulation within the gamut of our screen. To control attention, participants will be asked to respond to a rare orientation oddball Gabor stimulus, whilst refraining from responding to all other stimuli.

**3.7.1.5 Part C**

**31.** *Fundus camera photograph*. A fundus camera generates a 2D, high resolution image showing the optic disc, macula and blood vessels, and permits analysis of these anatomical features.

**32.** *Ultra-widefield scanning laser ophthalmoscopy*. An ultra-widefield scanning laser ophthalmoscope (UWF-SLO) captures 80% of the retina in an image.

**33.** *Optical coherence tomography (OCT).* OCT images the retinal nerve fibre layers (RNFL) and inner layers of the retina.

**34.** *OCT Angiography (OCT-A)*. OCT-A reveals the very small capillary level vessels in the retina which are not possible to image in any other way.

**35.** *Optical biometry*. Optical biometry yields the eye’s axial length - i.e. the distance from front to the back - a vital measure used to account for differences in individuals’ eyes and an important metric for the evaluation of retinal biomarkers.

**36.** *Automated perimetry*. Automated perimetry tests a participant’s visual field.

**37.** *Autorefraction*. Autorefraction measures a participant’s refractive error (i.e. how short- or long-sighted they are).

***3.7.2 Storage and analysis of samples***

Blood samples will be collected as part of the Part A protocol detailed in Section 3.2.4. A maximum of 31 blood samples will be collected in total as part of the study protocol. Control and BD Non-Lithium groups will have 30 samples collected. The ‘bipolar disorder on lithium’ group will have an additional sample collected to test lithium levels.

Sample collection

In the first instance, as noted above, one 2ml blood sample (‘bipolar disorder on lithium’ group only) and one 8ml blood sample will be taken during Part A. The 2ml sample will be used for lithium level analysis, and will be sent immediately for processing by the laboratory at the Royal Infirmary of Edinburgh. The 8ml sample will be processed and stored long-term at -80 degrees in University of Edinburgh facilities (The Institute of Genetics and Cancer, Western General Hospital) until use in future ethically approved research.

As described above, a total of 29 blood samples will be collected between the hours of 7pm and 6am across both Part A study nights for melatonin level analysis.

Sample storage

Blood samples for melatonin analysis will be centrifuged for plasma extraction and stored in freezers at -80C in the Clinical Research Facility, Royal Infirmary of Edinburgh. Samples will be stored according to the participant (i.e. all samples from one participant will be stored together in 1 box).

Blood samples for long-term storage (and future use in ethically approved studies) will be stored long-term at -80 degrees in University of Edinburgh facilities (The Institute of Genetics and Cancer, Western General Hospital) until use.

Sample processing

1 sample for lithium level analysis will be processed by the biochemistry laboratory at Royal Infirmary of Edinburgh. 1 sample for future ethically approved research (e.g. retinal organoid work (HELIOS-BD Workstream 1) which will be processed by our collaborators Lyle and Hilgen in Newcastle/Northumbria, subject to approvals). 29 samples for melatonin level analysis will be processed and analysed by NovoLytix GmbH, Switzerland. Samples will be analysed in patient batches to control for assay variance. Surplus sample from the analysis process will be destroyed.

### 3.8 Data collection

***3.8.1 Source data documentation***

Source data is defined as all information in original records and certified copies of original records or clinical findings, observations, or other activities in a clinical study necessary for the reconstruction and evaluation of the study. Source data are contained in source documents. Source documents are original documents, data and records where source data are recorded for the first time.

**3.8.1.1 Paper source documents**

•     Consent forms

•     Sleep diary

**3.8.1.2 Digital source documents**

•     Consent form

•     Electronic Case Report Form - includes procedural checklists and the following data collection instruments:

◦ Personal data and contact details
◦ Screening data (inclusion/exclusion criteria)
◦ Retrospective Assessment of the Lithium Response Phenotype (Alda) Scale score (standardised questionnaire of lithium response phenotype)
◦ Alcohol Use Disorders Identification Test (AUDIT) score (standardised questionnaire of alcohol use)
◦ Lifestyle information (standardised questionnaire)
◦ Past psychiatric history (standardised questionnaire)
◦ Medication history (standardised questionnaire)
◦ Pittsburgh Sleep Quality Index (PSQI) score (standardised questionnaire of sleep quality)
◦ Munich Chronotype Questionnaire (MCTQ) (standardised questionnaire of chronotype)
◦ Modified Seasonal Pattern Assessment Questionnaire (m-SPAQ) (standardised questionnaire of seasonality)
◦ WHODAS 2.0 Questionnaire score (standardised questionnaire of health and disability)

•     Blood sample analysis (melatonin levels)

•     Actigraphy data (electronic data collection) for 12 days prior to & including overnight CRF stays

•     Ambient light level data (electronic data collection) for 12 days prior to & including overnight CRF stays

•     Core Body Temperature (electronic data collection)

•     Psychophysical data: all psychophysical result files will contain single trial data indicating response accuracy at each stimulus level. Data will be stored as .txt files, to ensure compatibility.

•     Pupilometry: pupil size will be digitally recorded for each light exposure setting and stored in an open-source digital format.

•     Electroencephalography: Data will be recorded in Biosemi format but processed and stored using the default data format of the EEGlab toolbox for Matlab, which is a gold standard in digital EEG data processing.

***3.8.2 Case report forms***

The Case Report Forms are electronic and hosted on the Research Electronic Data Capture (REDCap) server software ( https://www.project-redcap.org/; RRID:SCR_003445) ( Harris *et al.*, 2009; Harris *et al.*, 2019). The REDCap software is accessed via a web application which allows users to enter and store data in a secure and intuitive user-friendly system. It is used around the world to securely gather research data.

 REDCap server software will be accessed using internet browsers on local University of Edinburgh network computers based in Kennedy Tower, University of Edinburgh laptops, remotely via the University of Edinburgh VPN and NHS Lothian computers located at the Clinical Research Facility, Royal Infirmary of Edinburgh. Only members of the research team will be able to register for a password-protected REDCap user account and have access to the study project on the server. Data will be extracted from the electronic CRFs as password-protected, encrypted excel spreadsheets, which will be stored on the University of Edinburgh DataStore, accessed via University of Edinburgh network computers based in the Division of Psychiatry, Kennedy Tower, Royal Edinburgh Hospital (University of Edinburgh).

### 3.9 Data management

***3.9.1 Data storage and access***

**3.9.1.1 Paper data**

All identifiable paper records (e.g. signed consent forms) will be stored in a locked filing cabinet in a locked office at the Kennedy Tower, Royal Edinburgh Hospital (University of Edinburgh). Only the research team members will have access to this, which includes investigators and collaborating groups who have been approved by the study investigators. University of Edinburgh policy regarding data management will be followed at all times ( https://www.ed.ac.uk/information-services/about/policies-and-regulations/research-data-policy). Following completion of the research project, identifiable paper records will be destroyed after 12 months.

**3.9.1.2 Digital data**

Screening & baseline visit

Digital identifiable data will be stored on secure REDCap server software and will be accessible for the duration of the study. The REDCap instance used for this study is run by Clinical Surgery, University of Edinburgh within the University of Edinburgh Virtual Machine architecture. It is physically secured (servers are physically located at the King’s Buildings campus, The University of Edinburgh, West Mains Road, Edinburgh, EH9 3JW, UK). All transmission and storage of web-based information by this online system is encrypted and was designed to be compliant with HIPAA-Security Guidelines.

Part A

Digital data from the eCelsius telemetric pill will be transmitted wirelessly to the eCelsius monitor in the CRF, and data from this will then be securely downloaded from the eCelsius monitor to the study computer at the Clinical Research Facility, via a direct USB connection. This data will be stored in a .csv spreadsheet, which will in turn be uploaded to the participant’s corresponding REDCap electronic CRF and downloaded to the University of Edinburgh study DataStore (controlled by Information Services at University of Edinburgh). Only authorised members of the research team will have access to the data store.

Digital data from the ActLumus light logger will be securely downloaded to a study computer at the Clinical Research Facility, via a direct USB connection. This data will be stored in a .csv spreadsheet, which will in turn be uploaded to the participant’s corresponding REDCap electronic CRF.

The NHS Lothian medical records of enrolled participants with bipolar disorder will be accessed by the study team to access any information relevant to the study, i.e., that entered at the Clinical Research facility and lithium levels, and then entered into the participant’s REDCap electronic CRF and downloaded to the study DataStore.

Digital data from the AX3 actigraph will be securely downloaded to a University of Edinburgh secure server, via a direct USB connection. This data will be stored in a .cwa file on the study DataStore.

After completion of each participant’s participation in the study, their electronic CRF data will be exported from the REDCap server and fileswithin an encrypted password protected folder on the study DataStore. Upon study completion all data stored using REDCap will therefore have been exported for processing and analysis purposes. All data will be deleted from REDCap 1 year after study completion.

Part B

Digital data from psychometric testing and EEG will be stored electronically in pseudonymised form in a secure folder on DataStore (controlled by Information Services at University of Edinburgh). Only authorized members of the research team will have access.

Part C

Digital data from retinal imaging will be stored electronically in pseudonymised form in a secure folder on DataStore (controlled by Information Services at University of Edinburgh). Only authorized members of the research team will have access.

All study data

Data held on the DataStore will be pseudonymised and the principles of data minimisation will always be followed. Only members of the research team will be able to access the data held on the DataStore, using a secure username and password. Any data which includes personal or identifiable information will additionally be encrypted and password protected.

Data .csv files will be accompanied by meta-data prepared in accordance with UK Data Service and Data Documentation Initiative guidelines. Documentation will include: a data dictionary (.csv file with rows for each variable and columns for: variable label, variable description, variable response scale codes, including missing data codes); a technical report that includes further background information on each variable (organised according to scales and subscales), including processing and further references; a study protocol detailing study design and data collection, with copies of all assessments and questionnaires annotated with variable labels included as appendices.

Personal data will be stored for 15 years after study completion on the University of Edinburgh DataVault. For one year following study completion, data will be held in pseudonymised format. After deletion of identifiable data (one year after study completion), the study dataset will effectively become anonymised. Anonymised digital data will be kept for a period of 14 years after the deletion of identifiable data.

No data will be shared out with the research team without explicit participant consent via the participant consent form. Anonymised data will be made available for download in line with open access principles. Anonymised data will be available for download via an Open Science Framework (OSF) project ( osf.io). This data will be available to the wider research community no later than 1 year after the completion of the project.

We will ensure published data (i.e. in academic manuscripts) does not contain any information that could disclose participant identity. Published results will be a descriptive analysis of aggregate results.

**3.9.1.3 Blood sample data**

For the melatonin level analysis, the samples will be sent from University of Edinburgh to NovoLytiX GmbH (CH-4108 Witterswil) and then extracted and measured with the NovoLytiX Melatonin RIA (RK-MEL2). They will be sent in a pseudonymised format, with the only identifier being the participant ID number. The results from this analysis will be entered into the Excel files sent in advance by a member of the research team to NovoLytiX GmbH, and subsequently sent back to the research team after completion of each batch of measurements. The raw data are archived anonymously at NovoLytiX GmbH and can be accessed by the principal investigators or other authorised third parties at any time during office hours (after prior registration).

For the lithium level analysis (BD on Lithium group only), results will be stored on the NHS TRAK database and will be linked to the participant’s CHI number. Only NHS staff will have access to these data.

***3.9.2 Transfer of data***

No data will be shared without explicit participant consent via the participant consent form. Participants will be asked for their GP and psychiatrist’s name, and for consent to inform them that they are taking part in the study, and of any notifiable results. This information will be posted or emailed securely to the GP.

***3.9.3 Data controller***

The joint data controllers are the University of Edinburgh and NHS Lothian.

***3.9.4 Data breaches***

Any data breaches will be reported to the University of Edinburgh ( dpo@ed.ac.uk) and NHS Lothian ( Lothian.DPO@nhslothian.scot.nhs.uk) Data Protection Officers who will onward report to the relevant authority according to the appropriate timelines if required.

### 3.10 Outcome measures

***3.10.1 Primary outcome measures***

**3.10.1.1 Part A**

Difference in absolute change in plasma melatonin levels in response to light stimulation protocol between the 3 study groups.

**3.10.1.2 Part B**

Difference in pupillometry, visual evoked potential, and chromatic discrimination/detection measures between the 3 study groups.

**3.10.1.3 Part C**

Retinal structure differences between the 3 groups assessed over time

***3.10.2 Secondary outcome measures***

**3.10.2.1 Part A**

To determine absolute differences in the following measures between the 3 study groups:

- ALDA scale (Bipolar Disorder groups only)
- PSQI total score for sleep quality
- MCTQ total score for chronotype
- MEQ total score for chronotype
- WHODAS 2.0 total score for global function
- m-SPAQ total score for seasonal sensitivity

**3.10.2.2 Part B**

Correlation of primary outcome measures with other, non-retinal measures acquired in the study (e.g. questionnaires, medical history), as well as with each other.

**3.10.2.3 Part C**

Secondary outcomes include cross-sectional and longitudinal differences in retinal vascular metrics; retinal layer thickness; small vessel metrics; eye biometrics; visual field metrics. These outcomes will be correlated with other, non-retinal measures acquired in the study.

### 3.11 Statistical analysis

***3.11.1 Proposed analyses***

**3.11.1.1 Part A analyses**

We will compute the area under the curve for the melatonin profile using the trapezoidal method and also compute the percentage difference between the healthy control group and the BD lithium therapy group, as well as between the healthy control group and the BD non-lithium treatment group. We will then use one-way ANOVA (or Kruskal-Wallis) with post-hoc analyses (Tukey) across the three groups, as well as undertaking general linear model analyses controlling for potential confounding variables such as age, sex, ethnicity, educational attainment, employment status, socioeconomic status, smoking status, alcohol use, sleep quality, chronotype, psychiatric history, medication use and score on the Alda lithium response scale. 

**3.11.1.2 Part B analyses**

Statistical analyses for the various measures (pupillary response, colour perception, spatiochromatic processing, visual evoked potentials) across the three groups will be performed using linear mixed effect models with healthy control group set as the baseline (i.e., zero) level for all the comparisons. Models will also be evaluated for the contribution of confounding factors, including age, sex, ethnicity, educational attainment, employment status, socioeconomic status, smoking status, alcohol use, sleep quality, chronotype, psychiatric history and medication use and score on the Alda lithium response scale.

**3.11.1.3 Part C analyses**

We will test the hypothesis that people with BD have significant differences in structural and functional measures of the retina and in the trajectory of change compared to controls. Additionally, we will assess whether individuals with BD who are taking lithium have an attenuated trajectory of change compared to those with BD on other medications. Student’s t-test and generalised estimation equations will be used to examine differences in retinal measures and psychophysical thresholds between the three participant groups. Spearman’s rank correlation coefficient will be used to investigate associations of retinal measures and psychophysical thresholds with other key parameters from the study data, including confounding demographic and clinical factors. Trajectory analysis will be used to assess changes in retinal parameters and psychophysical thresholds over time and how effective this approach is at stratifying participants.

### 3.12 Dissemination plan

The results of the HELIOS-BD study will be written up for publication in relevant scientific journals. Participants will receive a results summary letter once the study results are available, detailing the findings of the study and once again thanking them for their participation. Results from the study will additionally be summarised on the study website ( www.heliosbd.com).

### 3.13 Adverse events

Participants will be encouraged to contact the research team at any time should they experience any problems during or following their participation. All adverse events and adverse reactions will be recorded using the appropriate ‘ *Adverse Event or adverse reaction Log*’. Adverse events may occur in relation to procedures of Part A and Part B: telemetric pill ingestion; blood sampling/draws; light stimulation; and/or bilateral ocular application of pupil dilator medication. Serious adverse events (SAE’s) reporting will follow HRA procedure for REC notification and subsequent action, and will include any Serious Adverse Events (SARs) or Suspected SUSARs, as defined below.

***3.13.1 Definitions***

An adverse event (AE) is an untoward medical occurrence in a study participant.

An adverse reaction (AR) is any untoward and unintended response which is related to the outcome data collection for that participant. A serious adverse reaction (SAR) is any AR that:

- results in death of the clinical trial participant; is life threatening*;
- requires in-patient hospitalisation or prolongation of existing hospitalisation^;
- results in persistent or significant disability or incapacity;
- consists of a congenital anomaly or birth defect;
- results in any other significant medical event not meeting the criteria above.

A suspected unexpected serious adverse reaction (SUSAR) is any AR that is classified as serious and is suspected to be caused by the outcome data collection.

*Life-threatening in the definition of an SAE or SAR refers to an event where the participant was at risk of death at the time of the event. It does not refer to an event which hypothetically might have caused death if it were more severe.

^Any hospitalisation that was planned prior to randomisation will not meet seriousness criteria. Any hospitalisation that is planned post randomisation will meet the seriousness criteria unless it does not constitute an untoward medical occurrence (e.g. cosmetic elective surgery, social and/or convenience admission, etc.).

### 3.14 Good clinical practice

***3.14.1 Ethical conduct***

The study will be conducted in accordance with the principles of the International Conference on Harmonisation Tripartite Guideline for Good Clinical Practice (ICH GCP). Before the study can commence, all necessary approvals will be obtained and any conditions of approvals will be met.

***3.14.2 Informed consent***

The Investigator is responsible for ensuring informed consent is obtained before any study specific procedures are carried out. The decision of a participant to participate in clinical research is voluntary and should be based on a clear understanding of what is involved. Participants must receive adequate oral and written information – appropriate Participant Information and Informed Consent Forms will be provided. The oral explanation to the participant will be performed by the Investigator or qualified delegated person, and must cover all the elements specified in the Participant Information Sheet and Consent Form. The participant must be given every opportunity to clarify any points they do not understand and, if necessary, ask for more information. The participant must be given sufficient time to consider the information provided. It will be emphasised that the participant may withdraw their consent to participate at any time without loss of benefits to which they otherwise would be entitled. The participant will be informed and agree to their medical records being inspected by regulatory authorities and representatives of the Sponsor(s). The Investigator or delegated member of the study team and the participant will sign and date the Informed Consent Form(s) to confirm that consent has been obtained. The original will be signed in the Investigator Site File (ISF). The participant will receive a copy of the signed consent form and a copy will be filed in the participant’s medical notes and sent to their GP.

***3.14.3 Confidentiality***

All laboratory specimens, evaluation forms, reports, and other records must be identified in a manner designed to maintain participant confidentiality. All records must be kept in a secure storage area with limited access. Clinical information will not be released without the written permission of the participant. The Investigator and study site staff involved with this study may not disclose or use for any purpose other than performance of the study, any data, record, or other unpublished information, which is confidential or identifiable, and has been disclosed to those individuals for the purpose of the study. Prior written agreement from the Sponsor or its designee must be obtained for the disclosure of any said confidential information to other parties.

***3.14.4 Data protection***

All Investigators and study site staff involved with this study must comply with the requirements of the appropriate data protection legislation (including the General Data Protection Regulation and Data Protection Act) with regard to the collection, storage, processing and disclosure of personal information. Computers used to collate the data will have limited access measures via user names and passwords. Published results will not contain any personal data that could allow identification of individual participants.

***3.14.5 Study record retention***

Personal data will be stored for 15 years after study completion on the University of Edinburgh DataVault. For one year following study completion, data will be held in pseudonymised format. After deletion of identifiable data (one year after study completion), the study dataset will effectively become anonymised. Anonymised digital data will be kept for a period of 14 years after the deletion of identifiable data. When the minimum retention period has elapsed, study documentation will be destroyed with permission from the Sponsor.

***3.14.6 End of study***

The end of study is defined as the last participant’s last visit. The Investigators and/or the Sponsor(s) have the right at any time to terminate the study for clinical or administrative reasons. The end of the study will be reported to the REC, and R&D Office(s) and Sponsor(s) within 90 days, or 15 days if the study is terminated prematurely. The Investigators will inform participants of the premature study closure and ensure that the appropriate follow up is arranged for all participants involved. End of study notification will be reported to the Sponsor(s) via email to researchgovernance@ed.ac.uk. A summary report of the study will be provided to the REC within 1 year of the end of the study.

### 3.15 Ethical approvals

The HELIOS-BD study received a favourable ethical opinion from the South East Scotland Research Ethics Committee on 20/10/2023 (23/SS/0104). NHS Lothian R&D approvals were granted on 09/11/2023. The HELIOS-BD study is co-sponsored by the University of Edinburgh and NHS Lothian.

### 3.16 Ethical considerations of the research

1. Ensuring inclusive and representative recruitment:

Our recruitment methods are based on voluntary contact from interested individuals who have been informed of the study either through established research and mental health organisations, or via social media. However, it is known that particular identities are under-represented in medical research, with serious consequences for equitable healthcare. In particular, people from minoritised ethnic groups, people at socioeconomic disadvantage and people with minoritised sexualities and gender identities are under-represented and under-served by research. The reasons for this are many and varied. In this study, we aim to achieve a representative and diverse study population, with equal representation of men and women. We will advertise the study widely, and we will recruit thoughtfully to ensure our sample is representative of the bipolar disorder population in Scotland. We will also provide reimbursement for caring costs (e.g. childcare) to remove barriers to participation for those with caring responsibilities.

2. Preventing undue incentivisation due to participation payments:

Study participants will be reimbursed for their time and travel expenses. However, it is important that financial benefits do not inappropriately incentivise individuals to participate in the research when it is not in their best interests. The participation payment will therefore be advertised in a neutral tone in any study advert materials and in the Participant Information Form. We recognise that offering payment for study participation may increase the possibility of dishonesty from people who want to participate in the research, in order to increase their chances of enrolment. We will address this by using NHS-linked recruitment methods and by corroborating information with medical notes where necessary.

3. Risks and adverse events associated with study procedures:

Some of the study procedures carry risks for adverse events; for example, participants may experience short-term discomfort during the study procedures required for this study. Associated risks will be minimised by ensuring that experienced staff are appropriately trained to conduct the procedures, and that participants are fully informed of all procedures. Participants will be instructed to inform the research team of any health issues or concerns which arise during the study, to make sure they are appropriately cared for. A wellbeing follow-up call is scheduled for all participants 3 days following their final overnight stay to make sure there are no negative impacts of the light delivery protocol.

Further to this, there is a small risk that incidental findings may be identified on the retinal images which may require further investigation. We anticipate the rate of significant or urgent findings to be <1%. Each participant will be told of this risk, as well as it being stated on the participant information sheet. Should we observe unusual signs in the images that might signs of eye problems, anonymised images will be reviewed by the ophthalmologist on our team for checking of incidental findings. If we were to observe any such findings then an appropriate member of the study team would discuss this with the participant and inform their GP so that appropriate further tests and treatments could be arranged as necessary.

4. Making sure the research is relevant and acceptable to people with bipolar disorder:

It is important that the research we conduct is relevant and acceptable to people with bipolar disorder. We are in ongoing consultation with the HELIOS-BD Lived Experience Advisory Panel (LEAP) and volunteers from Bipolar Scotland to make sure this is the case. The study design has already benefited from input from people with lived experience of bipolar disorder.

## 4 Study Status

Study is active and recruiting participants.

## 5 Conclusion

HELIOS-BD tests an important and still unresolved hypothesis on bipolar disorder light hypersensitivity and the mechanism of action of lithium at both cellular and systems levels. With this ambitious project, we seek to investigate visual and non-visual responses to light, and associated structural and functional retinal changes, in a bipolar disorder population compared to control participants. Further, the study will test whether lithium acts to correct any altered responses. The results from this project have considerable translational potential and will help us to further understand circadian dysregulation in bipolar disorder, as well as potentially provide insight into therapeutic pathways to prevent acute mood episodes.

## Data Availability

No data are associated with this article.
