# Supplementary material for: Investigating light sensitivity in bipolar disorder (HELIOS-BD)
Source: Wellcome Open Res. 2024 Oct 18;9:64. Originally published 2024 Feb 19. [Version 2] doi: 10.12688/wellcomeopenres.20557.2 (PMC11074697; doi:10.12688/wellcomeopenres.20557.2)
Supplement: Peer review history

# Reviewer report: Reviewer response for version 2

**DOI:** 10.21956/wellcomeopenres.25555.r107242

**Published:** 2024-11-12

**Authors:** Angus C. Burns

**Affiliations:** 1 Program in Medical and Population Genetics, Broad Institute, Cambridge, Massachusetts, USA; 2 Brigham and Women’s Hospital, Boston, USA; 3 Division of Sleep and Circadian Disorders, Harvard Medical School Department of Medicine, Boston, Massachusetts, USA

The authors have put together a novel and exciting protocol that aims to examine the role of light exposure and light sensitivity in the aetiology of bipolar disorder. This project is well motivated by prior literature on a putative role for non-visual light sensitivity in bipolar disorder and seeks to identify plausible mechanisms at the level of the retina. The project involves three study arms designed to examine whether bipolar disorder patients exhibit (A) an enhanced non-visual response of the circadian clock to light as measured by melatonin suppression, (B) aberrant visual, cortical and pupillary responses to light of different wavelengths designed to target different photoreceptor systems and (C) distinct microstructural changes in the retina, as compared to healthy controls with no history of a psychiatric disorder. As an additional layer of investigation and analysis, the authors will examine whether lithium treatment will act as a moderator of hypothesised differences between bipolar disorder cases and controls, such that those treated with lithium are closer to controls in phenotypic presentation. I note that this protocol has undergone a revision in response to reviews from several colleagues with updates to inclusion/exclusion criteria and pupillometry methods. The proposed methodology is overall well designed to address the proposed aims and I applaud the involvement of individuals with lived experience of bipolar disorder. However, I have some further questions and comments in particular relating to the melatonin suppression protocol. 

3.4 Study Design

 You state that “this is an experimental study”, which I don’t think is correct for your main aims and hypotheses which relate to differences in melatonin suppression, pupillary responses and retinal microstructure between diagnosis groups. As the case-control status of bipolar *versus *healthy control participants is not manipulated or randomised this study is perhaps better described as an observational case-control study with experimental manipulation within groups (e.g. light exposure manipulation) or a quasi-experimental study. This also applies to the lithium moderation hypothesis which as far as I can tell is not going to be randomized and/or manipulated. I am noting that there is a reference to randomisation in 3.13.1 Definitions, is this related to the actual study procedures?

3.4.4.1 Baseline study visit

 Just noting to include the MEQ here and in 3.7.1.2 Baseline study visit as a measure of chronotype which is referred to in 3.10.2.1 Part A. I think it is good to have both since they tap into different dimensions of chronotype.

Part A: Overnight experimental assessments

1. It is great that the authors have included a baseline/dark control for this study, and I find the use of a core-body temperature measurement interesting. The aim of this is to time the administration of the light exposure on Study Day 2 relative to the CBT nadir, i.e., standardizing the circadian phase of administration. This is a great idea in principle however I am concerned by the fact that participants are free to sleep from 10pm to 6am. Sleep causes a dramatic masking of the core body temperature rhythm (see Figures 3 and 5 in this paper ^1^). As such, it is gold-standard practice to measure CBT under constant routine procedures with participants maintaining wakefulness in order to accurately measure intrinsic timing. Under the current protocol, you are likely to observe different CBT profiles to the true rhythm. Can the authors provide a rationale for the current methodology? Is it possible to switch this to a constant routine to ensure accurate CBT rhythm measurement?
2. Following from this, the masking effect of sleep differs by the circadian phase of sleep onset. I note that the sleep onset times are fixed for both groups and not based on habitual sleep onset and offset. This means that the circadian phase of sleep onset will likely systematically differ between-groups given a proposed circadian delay amongst bipolar patients. This may result in a systematic bias in the estimate of CBT minima between groups and result in the timing of the light exposure at different circadian phases between groups. I think the authors will want to avoid this as it could result in the occlusion of true group differences.
3. Relating to the above points, I note that you propose to use the mid-point of sleep based on sleep diaries for the 10 days prior to time the light exposure if the CBT monitoring fails to detect a minimum. This is a good idea in general and I think could be a more accurate alternative procedure to the CBT measurement approach if you are not interested in running a constant routine. In that case, I would suggest imposing a self-selected fixed sleep-wake schedule for the 10 days prior to lab visit (to ensure stable entrainment) and then time the light exposure based on the midpoint of sleep. This should be checked with objective actigraphy. Currently, I believe the participants are on a free schedule prior to the lab visits. This may not be ideal due to the large variability of sleep-wake timing behaviour in free-living humans (which is potentially pronounced in bipolar disorder) and may limit the utility of a sleep-diary based midpoint for timing the light exposure. Alternatively, one could run a constant routine to ensure accurate measurement of the CBT rhythm to time the light exposure.
4. I am wondering if based on the power analysis you have selected a light level for the exposure? Further, I was wondering why a 30-minute exposure only was selected, as there are temporal dynamics to melatonin suppression that could be interesting as well.

Is the study design appropriate for the research question?

Partly

Is the rationale for, and objectives of, the study clearly described?

Yes

Are sufficient details of the methods provided to allow replication by others?

Yes

Are the datasets clearly presented in a useable and accessible format?

Not applicable

Reviewer Expertise:

Circadian rhythms, sleep, light exposure, melatonin suppression, genetics, psychiatric health.

I confirm that I have read this submission and believe that I have an appropriate level of expertise to confirm that it is of an acceptable scientific standard, however I have significant reservations, as outlined above.

## References

[1] : Contribution of the circadian pacemaker and the sleep homeostat to sleep propensity, sleep structure, electroencephalographic slow waves, and sleep spindle activity in humans. *J Neurosci* .1995;15(5 Pt 1) : 10.1523/JNEUROSCI.15-05-03526.1995 3526-38 10.1523/JNEUROSCI.15-05-03526.1995 7751928 PMC6578184

# Reviewer report: Reviewer response for version 2

**DOI:** 10.21956/wellcomeopenres.25555.r107248

**Published:** 2024-11-11

**Authors:** Joey W. Y. Chan

**Affiliations:** 1 The Chinese University of Hong Kong, Hong Kong, Hong Kong

This is a timely and important research study to investigate the largely unknown impact of light sensitivity, retinal functions, and lithium in patients with bipolar disorder. The study seeks to unveil the potential neurobiological mechanism of light contributing to circadian disruptions in bipolar disorder and the therapeutic mechanism of lithium. This is a relatively large study incorporated both state-of-the-art visual/ retinal assessments and in-depth circadian measurements. The rationale and objectives of the study are well-defined. There are established patient networks for the recruitment and early involvement of patients to delineate an acceptable research protocol.

 I have some minor queries and comments based on the version2:

**Background 2.2:** “A hypo- or hyper-sensitive response to light within the photoreceptive pathway can therefore result in a shifted circadian phase and dysregulation of biological rhythms.”

 In healthy young adults, a more pronounced PIPR was associated with a more delayed sleep (van der Meijden et al., 2016). On the other hand, we found that a larger PIPR correlated with an earlier acrophase of the actigraphic rest-activity rhythm (Chan et al., 2024). While the light

 sensitivity may influence the melatonin levels, the shift of the circadian phase would also be dependent on the light exposure pattern. It is very thoughtful to measure continuous light exposure in the study. How would the light exposure data be analysed?

**3.4.4.3 Part B: Retinal assessments: Session 1: Pupillometry**

 It would be helpful if the authors can provide greater details about the light stimulus, pupillometric protocol and definition of PIPR in the methods. Given the circadian modulation of PIPR (Munch et al., 2012; Zele et al., 2011), would PIPR be conducted according to the individual circadian time in this study? Or constrained to a certain time of the day, as in other studies (Bullock et al., 2019)?

**3.6.2 Inclusion criteria: BD non-lithium participant group:**

 The difference between the BD-lithium and BD non-lithium group is the absence of lithium treatment over the past six months prior to entry of the study. Other psychotropics, e.g. sodium valproate (Hallam et al., 2005), SSRI (McGlashan et al., 2018), have also be shown to influence light sensitivity, would the effects of these medications on light sensitivity be accounted for in the analysis?

**3.6.3 Exclusion criteria:** All participants: blindness, visual impairment, existing eye disease such as glaucoma or age-related macular degeneration, retinal surgery etc Presence of cataract may reduce the amount of light reaching the retina in the pupillary response measurements. Given that comprehensive eye assessment is available, it would be helpful to measure lens opacity if cataract is not an exclusion.

Is the study design appropriate for the research question?

Yes

Is the rationale for, and objectives of, the study clearly described?

Yes

Are sufficient details of the methods provided to allow replication by others?

Yes

Are the datasets clearly presented in a useable and accessible format?

Not applicable

Reviewer Expertise:

Bright light therapy, circadian rhythms, pupillary response, affective disorders

I confirm that I have read this submission and believe that I have an appropriate level of expertise to confirm that it is of an acceptable scientific standard.

## References

[1] : Traits related to bipolar disorder are associated with an increased post-illumination pupil response. *Psychiatry Res* .2019;278: 10.1016/j.psychres.2019.05.025 35-41 10.1016/j.psychres.2019.05.025 31136914

[2] : Attenuated melanopsin-mediated post-illumination pupillary response (PIPR) is associated with reduced actigraphic amplitude and mesor in older adults. *Sleep* .2024; 10.1093/sleep/zsae239 10.1093/sleep/zsae239 39383299

[3] : Effect of sodium valproate on nocturnal melatonin sensitivity to light in healthy volunteers. *Neuropsychopharmacology* .2005;30(7) : 10.1038/sj.npp.1300739 1400-4 10.1038/sj.npp.1300739 15841104

[4] : The SSRI citalopram increases the sensitivity of the human circadian system to light in an acute dose. *Psychopharmacology (Berl)* .2018;235(11) : 10.1007/s00213-018-5019-0 3201-3209 10.1007/s00213-018-5019-0 30219986

[5] : Circadian and wake-dependent effects on the pupil light reflex in response to narrow-bandwidth light pulses. *Invest Ophthalmol Vis Sci* .2012;53(8) : 10.1167/iovs.12-9494 4546-55 10.1167/iovs.12-9494 22669721

[6] : Individual Differences in Sleep Timing Relate to Melanopsin-Based Phototransduction in Healthy Adolescents and Young Adults. *Sleep* .2016;39(6) : 10.5665/sleep.5858 1305-10 10.5665/sleep.5858 27091519 PMC4863220

[7] : The circadian response of intrinsically photosensitive retinal ganglion cells. *PLoS One* .2011;6(3) : 10.1371/journal.pone.0017860 e17860 10.1371/journal.pone.0017860 21423755 PMC3056772

# Reviewer report: Reviewer response for version 2

**DOI:** 10.21956/wellcomeopenres.25555.r107247

**Published:** 2024-10-28

**Authors:** Oleg Kovtun

**Affiliations:** 1 Vanderbilt University, Nashville, USA

The protocol by Roguski *et al*. is a part of broader effort funded by the Wellcome Trust to investigate whether light hypersensitivity causes circadian rhythm disruption in bipolar disorder. The three-pronged longitudinal study protocol outlines a rigorous plan to evaluate the contribution of retinal pathophysiology to hypothesized light hypersensitivity in BD patients by examining (i) light-induced melatonin suppression, (ii) retinal function, and (iii) retinal microstructure, compared to healthy controls. In addition, the protocol seeks to determine whether the mood stabilizer lithium reduces heightened light sensitivity and mitigates retinal pathophysiological changes. 

 The rationale for the protocol is supported by a broad consensus that circadian disturbances are a hallmark feature across various BD phenotypes. Light (and its seasonal patterns) serves as a critical cue for entraining circadian rhythms and has emerged as a powerful therapeutic tool (e.g., bright-light therapy (BLT) and blue-light blocking sunglasses) to restore natural circadian rhythms, thereby reducing symptoms and functional impairment in BD. Several studies suggested that hypersensitivity to light may be a trait marker for BD, which is in excellent agreement with the hypothesis put forth by Roguski *et al*. Furthermore, investigating the link between retinal physiology and light sensitivity in BD addresses several central questions in the future research agenda from the International Society for Bipolar Disorders (ISBD) Chronobiology Task Force (CTF) [https://doi.org/10.1111/bdi.13165].

 The protocol is meticulously developed and organized:

- The mechanistic basis for investigation is clearly defined in the introduction;
- Lived experience is incorporated into the protocol and remains central throughout the study, from the participant screening stage to data interpretation to results dissemination;
- Inclusion/exclusion criteria are appropriate and clearly defined;
- A structured clinical assessment is in place to confirm a diagnosis of BD and clinical euthymia prior to enrollment. The assessment includes complementary questionnaires to ensure rigor;
- The on-site experimental assessments are described in appropriate detail and were designed to minimize the burden and risk of adverse events;
- Adequate data management protocols are in place to protect participant’s sensitive personal data;
- Ethical approvals were granted in accordance with the principles of good clinical practice.

The authors fully addressed all concerns raised by the reviewers in the first round. However, I do have several minor comments:

1. In section 2.3, Roguski *et al*. describe the observed seasonal patterns in mood episode-related hospital admissions and suicide attempts in BD. Recognizing the importance of seasonality, the authors included a Modified Seasonal Pattern Assessment Questionnaire (m-SPAQ) in the baseline assessment. Beyond that, it is not clear how seasonality is incorporated into Part A overnight experimental assessments and subsequent data analysis. Yes, there is a plan to conduct Part A between the months of September-April inclusive to avoid the effects of too much light at night during the summer months. However, is there a plan to assign study participants into Fall, Winter, Spring subgroups to capture seasonality effects?
2. The association of BD with medical comorbidities is well-documented. Recent evidence also suggests higher incidence rates of retinal diseases in BD patients compared to healthy controls [https://doi.org/10.1111/pcn.13326]. Additionally, lithium is associated with ophthalmic adverse effects, including exophthalmos, abnormal eye movements, photophobia, and abnormal tear film [https://doi.org/10.1097/YIC.0000000000000295]. Roguski *et al*. anticipate that incidental findings may be identified on the retinal images, with the anticipated rate of <1% - is there a reference to support this number? Appropriately, the medical exclusion criteria include visual impairments, existing eye disease (glaucoma or age-related macular degeneration), and previous surgery on the retina. However, have the authors considered assessing medical comorbidities that are risk factors for retinal health (e.g., type 2 diabetes, CVD, hypertension) as potential confounding variables?
3. Section 3.10.2.1. Part A lists MEQ (Morningness-Eveningness Questionnaire?) total score for chronotype as a secondary outcome measure, but I couldn’t find it anywhere else in the protocol.
4. Section 3.16.3 (last paragraph): “ *Should we observe unusual signs in the images that might signs of eye problems*,…” – might indicate signs?

Overall, this is a comprehensive and rigorously structured protocol for an important and much-needed study in BD etiology. I look forward to seeing the results.

Is the study design appropriate for the research question?

Yes

Is the rationale for, and objectives of, the study clearly described?

Yes

Are sufficient details of the methods provided to allow replication by others?

Yes

Are the datasets clearly presented in a useable and accessible format?

Not applicable

Reviewer Expertise:

Seasonality in mood disorders, circadian rhythm, accelerometry, digital mental health

I confirm that I have read this submission and believe that I have an appropriate level of expertise to confirm that it is of an acceptable scientific standard.

# Reviewer report: Reviewer response for version 1

**DOI:** 10.21956/wellcomeopenres.22755.r76485

**Published:** 2024-05-06

**Authors:** Karen Hallam

**Affiliations:** 1 RMIT University, Melbourne, Victoria, Australia

The rationale for this research is strong and circadian rhythms have long been an identified marker of the illness and disturbances of these a key treatment focus for clinicians. The authors have identified several key papers that span over 50 years now, with many of these identifying similar trends in melatonin suppression.

 IN relation to the effects of lithium carbonate and other lithium preparations on light sensitivity, there is a strong rationale for investigating this response. It is worth noting that the authors have not included some of our previous research in healthy volunteers showing a direct effect of lithium carbonate on reducing melatonin sensitivity to light in health volunteers, a finding that implies one potential physiological impact of lithium that may be involved in its management of bipolar disorders (see Hallam et al, 2005(Ref 2)). This further highlights the need to better understand and potential associate lithium levels with light sensitivity in large scale studies such as that being proposed. It is notable that we identified similar (albeit weaker) changes in melatonin sensitivity to light with sodium valproate treatment (Hallam et al., 2005 (Ref 3)).

 The recruitment approach is excellent and the capacity to use databases like SHARE provide a new method to manage selection biases in research recruitment. This will likely deliver data on more participants across functional levels, something that may further the research from previously hospital recruited participant groups. In terms of the study aims and methodology, the aims are clearly defined and there is a sound rationale. The study is large and has a well-developed methodology. I would suggest considerations for the authors for their methodology to improve the outcomes or to consider in more depth below.

 In terms of measurement tools, confirmation of their diagnosis outside of clinical interview seems to have been excluded. It would be suggested that a formal screening questionnaire (e.g. the SCID) is utilised to standardise the assessment process. This may be particularly useful for differentiating bipolar type I and II populations. Notably, when assessing lifestyle factors, the team may want to investigate caffeine use (not covered in the AUDIT), which falls outside the AUDIT but has a clear impact on sleep quality for many people and potentially populations with BPAD (see Frigerio S,et.al., 2021(Ref 1)). It would also be logical to screen for other substance use.

 In relation to screening, it would typically be expected that the investigators would assess manic and/or hypomanic symptoms at the time of testing and study inclusion. Typically, we would encourage the use of the Young Mania Rating Scale (YMRS) or similar to 1. Ensure someone can consent to the research and 2. To identify their level of active manic/hypomanic symptoms. This should be re-administered for any further testing sessions.

 The procedure seems appropriate and scientifically rigorous, utilising highly sensitive measurement approaches that would not have been previously available to researchers (e.g. temperature pill). Adding the details of the brightness of the full spectrum white light (e.g. using a luxometer) would be a helpful addition to assess against past studies.

 Finally, in terms of methodology, I am unsure of why the detailed ocular investigations follow the overnight melatonin measurement. It would make more sense for these tests to be conducted first to ensure the individual is appropriate for the study as any deficits in these measures may impact the participants results.

 This is a fascinating and rigorously developed study with a large population that may finally answer the question around melatonin light sensitivity and its role in bipolar disorders. The research is important, both for the basic science it is uncovering as well as its clinical implications for interventions. I wish the research team the very best for this important project.

Is the study design appropriate for the research question?

Yes

Is the rationale for, and objectives of, the study clearly described?

Yes

Are sufficient details of the methods provided to allow replication by others?

Yes

Are the datasets clearly presented in a useable and accessible format?

Not applicable

Reviewer Expertise:

Endophenotype research and application of biological theories to clinical interventions, particularly in the area of mood disorders and chronic stress.

I confirm that I have read this submission and believe that I have an appropriate level of expertise to confirm that it is of an acceptable scientific standard.

## References

[1] : The impact of caffeine consumption on clinical symptoms in patients with bipolar disorder: A systematic review. *Bipolar Disord* .2021;23(3) : 10.1111/bdi.12990 241-251 10.1111/bdi.12990 32949106

[2] : Low doses of lithium carbonate reduce melatonin light sensitivity in healthy volunteers. *Int J Neuropsychopharmacol* .2005;8(2) : 10.1017/S1461145704004894 255-9 10.1017/S1461145704004894 15850501

[3] : Effect of sodium valproate on nocturnal melatonin sensitivity to light in healthy volunteers. *Neuropsychopharmacology* .2005;30(7) : 10.1038/sj.npp.1300739 1400-4 10.1038/sj.npp.1300739 15841104

# Response

**Published:** 2024-10-01

**Authors:** Amber Roguski

**Affiliations:** Division of Psychiatry, The University of Edinburgh, Edinburgh, Scotland, UK

We thank the reviewer for their thoughtful and comprehensive review of the HELIOS-BD protocol, and for their fundamental contributions to the field. Our responses are as follows, and we respond specifically to the below points for clarification or to indicate an update to the manuscript.

 1. It is worth noting that the authors have not included some of our previous research in healthy volunteers showing a direct effect of lithium carbonate on reducing melatonin sensitivity to light in health volunteers, a finding that implies one potential physiological impact of lithium that may be involved in its management of bipolar disorders (see Hallam et al, 2005(Ref 2)). The protocol introduction has been updated to include this study, which provides important evidence for a role for lithium is regulation of circadian and melatonergic signalling. Thank you for highlighting this oversight.

 2. In terms of measurement tools, confirmation of their diagnosis outside of clinical interview seems to have been excluded. It would be suggested that a formal screening questionnaire (e.g. the SCID) is utilised to standardise the assessment process.  All potential participants with bipolar have their bipolar diagnosis confirmed by a psychiatrist during the screening appointment using the SCID. The responses to all SCID questions are stored for later analysis, for example, to stratify participants according to bipolar Type and polarity of symptoms (as suggested by the reviewer).

 3. Notably, when assessing lifestyle factors, the team may want to investigate caffeine use (not covered in the AUDIT), which falls outside the AUDIT but has a clear impact on sleep quality for many people and potentially populations with BPAD (see Frigerio S,et.al., 2021(Ref 1)). It would also be logical to screen for other substance use. Caffeine use is not covered during the screening appointment, but we do gain detailed insight into caffeine consumption during the Part A procedure when participants fill out a consensus sleep diary for 12 days. This asks for details relating to consumption of caffeine, alcohol and medications. During the screening appointment, all potential participants are questioned relating to their alcohol and substance use and are not eligible to participate in the study if harmful or dependent use is indicated.

 4. In relation to screening, it would typically be expected that the investigators would assess manic and/or hypomanic symptoms at the time of testing and study inclusion. -> Euthymic mood state is determined during the screening procedure, using the Structured Clinical Interview for DSM Disorders (SCID), administered by one of the research team (who has been trained in the SCID use). The majority of study activities occur within a short window following the screening appointment and euthymic mood state is assumed at these appointments. Participants are instructed to inform the study team if they experience a mood episode at any point during their participation. Eligibility criteria, including mood symptoms, are assessed by trained members of the research team prior to all study visits.  

 5. I am unsure of why the detailed ocular investigations follow the overnight melatonin measurement. It would make more sense for these tests to be conducted first to ensure the individual is appropriate for the study as any deficits in these measures may impact the participants results. The naming of the Part A, B and C appointments does not reflect their ordering in practice. Typically, participants attend the Baseline appointment and then their first Part C (retinal imaging) appointment in quick succession. We can therefore very quickly identify any previously undiscovered ocular issues in a timeframe very close to the enrolment of the participant. Appropriate action can then be taken (e.g. exclusion of the participant from further study activities). The Part A and Part B appointments are scheduled in the following months according to availability of the participant and the research team. Only Part C 9- and 18-month follow-up appointments must be scheduled within a strict timeframe – the remaining appointments are flexible in their scheduling.

# Reviewer report: Reviewer response for version 1

**DOI:** 10.21956/wellcomeopenres.22755.r76484

**Published:** 2024-03-20

**Authors:** Yuichi Esaki

**Affiliations:** 1 Fujita Health University School of Medicine, Toyoake, Japan

Reviewer comment:

 Light manipulation, including bright light therapy and dark therapy, is widely used for bipolar disorder in clinical practice. Nevertheless, light sensitivity in bipolar disorder is debatable. Also, lithium is the first-line treatment in bipolar disorder, but the underlying mechanisms remain unclear. Therefore, clarifying these is very important in advancing the mechanisms and treatments for bipolar disorder.

 This study protocol investigates an important and still unresolved hypothesis on bipolar disorder light hypersensitivity and the mechanism of action of lithium. This study provides three parts: Part A: Non-visual responses, including sleep/wake cycle, dim light melatonin onset, and core body temperature, Part B: Retinal sensitivity, including visual, cortical, and pupillary, Part C: Retinal imaging. Additionally, this study tests not only comparing patients with bipolar disorder and control participants but also comparing people with bipolar disorder either taking, or not taking, lithium.

 This is a very interesting study, and I cannot wait to see the results.

 I have some comments.

 1. Figure 2.

 Do the participants take part in all parts, including Part A, B, and C? Or do they take part in only one part?

 2. Page 9. 3.4.4.3 Part B: Retinal assessments

 “Experimental assessments will be conducted at the Colour and EEG laboratories (Dugald Stewart Building, University of Edinburgh) between the months of September–April inclusive to avoid the effects of too much light at night during the summer months.”

 Page 11. 3.5 Data collection/participation windows

 “Baseline, Part B and Part C study visits and data collection will take place throughout the year.”

 Which is correct? Will Part B study data collection take place between the months of September–April or throughout the year?

 3. Page 11. 3.6 Study population

 This is a longitudinal study. What happens if a participant taking lithium stops taking it during follow-up? Drop out? Or will the data be included in the analysis? Also, what happens if a participant not taking lithium starts taking it during follow-up?

 4. Page 12. BD Lithium participant group

 Who and how determine clinically euthymic of the participants? For example, the International Society for Bipolar Disorders Task Force recommends a MADRS or YMRS score of <8 points to indicate symptomatic remission of bipolar depression or mania, respectively (Tohen et al., 2009). Do the authors assess mood symptoms at the baseline assessment?

 5. Table 1.

 To facilitate understanding of Table 1 and the manuscript, I suggest that each assessment item be numbered to match the manuscript.

 6. Page 15.

 Regarding the AX3 actigraphy device (Axivity, UK) and ActLumus light logger (Condor Instruments, Brazil), are these devices validated in the previous studies? If so, please add the references.

Is the study design appropriate for the research question?

Yes

Is the rationale for, and objectives of, the study clearly described?

Yes

Are sufficient details of the methods provided to allow replication by others?

Yes

Are the datasets clearly presented in a useable and accessible format?

Yes

Reviewer Expertise:

Psychiatry, Chronobiology, in particular light exposure

I confirm that I have read this submission and believe that I have an appropriate level of expertise to confirm that it is of an acceptable scientific standard.

# Response

**Published:** 2024-04-18

**Authors:** Amber Roguski

**Affiliations:** Division of Psychiatry, The University of Edinburgh, Edinburgh, Scotland, UK

We thank the reviewer for their careful consideration of the HELIOS-BD study and their thoughtful questions and suggestions. Our responses are as follows, and we will make amendments to the manuscript in due course to provide further clarification on the below points.

**Reviewer Comment:**

 1. Figure 2.

 Do the participants take part in all parts, including Part A, B, and C? Or do they take part in only one part? -

 A) Participants are expected to complete all parts of the study (Baseline, Part A-C) and are recruited onto the study under that expectation.

**Reviewer Comment:**

 2. Page 9. 3.4.4.3 Part B: Retinal assessments

 “Experimental assessments will be conducted at the Colour and EEG laboratories (Dugald Stewart Building, University of Edinburgh) between the months of September–April inclusive to avoid the effects of too much light at night during the summer months.”

 Page 11. 3.5 Data collection/participation windows

 “Baseline, Part B and Part C study visits and data collection will take place throughout the year.”

 Which is correct? Will Part B study data collection take place between the months of September–April or throughout the year?

 A) Thank you for highlighting this inconsistency – Baseline, Part B and Part C appointments will take place throughout the year. Only Part A appointments are stopped during the months of May – August (inclusive). We will amend the manuscript accordingly.

**Reviewer Comment:**

 3. Page 11. 3.6 Study population

 This is a longitudinal study. What happens if a participant taking lithium stops taking it during follow-up? Drop out? Or will the data be included in the analysis? Also, what happens if a participant not taking lithium starts taking it during follow-up?

 A) Inclusion criteria for the two bipolar groups state that participants must have been taking lithium consistently for the past 6 months (bipolar on lithium group) or have not taken lithium for the past 6 months (bipolar not on lithium group). A change in lithium medication, either stopping or starting, would therefore serve as an exclusion criterion and make the participant in question ineligible for continued participation. If a participant therefore has a lithium-related medication change, they are withdrawn from the study. Their data up to the change would be kept and included in analyses.

**Reviewer Comment:**

 4. Page 12. BD Lithium participant group

 Who and how determine clinically euthymic of the participants? For example, the International Society for Bipolar Disorders Task Force recommends a MADRS or YMRS score of <8 points to indicate symptomatic remission of bipolar depression or mania, respectively (Tohen et al., 2009). Do the authors assess mood symptoms at the baseline assessment?

 A) Euthymic mood state is determined during the screening procedure, using the Structured Clinical Interview for DSM Disorders (SCID), administered by one of the research team (who has been trained in the SCID use). The majority of study activities occur within a short window following the screening appointment and euthymic mood state is assumed at these appointments. Participants are instructed to inform the study team if they experience a mood episode at any point during their participation. Eligibility criteria, including mood symptoms, are assessed prior to all study visits.

**Reviewer Comment:**

 5. Table 1.

 To facilitate understanding of Table 1 and the manuscript, I suggest that each assessment item be numbered to match the manuscript.

 A) Thank you for this suggestion – we will amend the manuscript accordingly.

**Reviewer Comment:**

 6. Page 15.

 Regarding the AX3 actigraphy device (Axivity, UK) and ActLumus light logger (Condor Instruments, Brazil), are these devices validated in the previous studies? If so, please add the references. 

 A)  AX3 device has been validated in several adult populations for sleep and activity measures. ActLumus device has not yet been clinically validated. We will amend the manuscript accordingly to include references regarding the AX3 validation studies.
